# Stiff extracellular matrix activates the transcription factor ATF5 to promote the proliferation of cancer cells

**DOI:** 10.1016/j.isci.2025.112057

**Published:** 2025-02-17

**Authors:** Seiichiro Ishihara, Atsushi Enomoto, Akihiro Sakai, Tadashi Iida, Shoichiro Tange, Noriyuki Kioka, Akihiro Nukuda, Ayaka Ichikawa Nagasato, Motoaki Yasuda, Takashi Tokino, Hisashi Haga

**Affiliations:** 1 Department of Advanced Transdisciplinary Sciences, Faculty of Advanced Life Science, Hokkaido University, Sapporo, Hokkaido 060-0810, Japan; 2 Department of Pathology, Nagoya University Graduate School of Medicine, Nagoya, Aichi 466-8550, Japan; 3 Department of Gastroenterology and Hepatology, Nagoya University Graduate School of Medicine, Nagoya, Aichi 466-8550 Japan; 4 Department of Medical Genome Sciences, Cancer Research Institute, Sapporo Medical University School of Medicine, Sapporo, Hokkaido 060-8556, Japan; 5 Division of Applied Life Sciences, Graduate School of Agriculture, Kyoto University, Kyoto 606-8502, Japan; 6 Institute for Integrated Cell-Material Sciences (iCeMS), Kyoto University, Kyoto 606-8501, Japan; 7 Transdisciplinary Life Science Course, Faculty of Advanced Life Science, Hokkaido University, Sapporo, Hokkaido 060-0810 Japan; 8 Department of Oral Molecular Microbiology, Division of Oral Pathobiological Science, Faculty of Dental Medicine and Graduate School of Dental Medicine, Hokkaido University, Sapporo, Hokkaido 060-8586, Japan

**Keywords:** Biophysics, Cancer, Microenvironment

## Abstract

Cancer tissues are stiffer than normal tissues. Carcinogenesis stiffens the extracellular matrix (ECM) of cancerous tissues, to which cancer cells respond by activating transcription factors, such as YAP/TAZ, Twist1, and β-catenin, which further elevate their malignancy. However, these transcription factors are also expressed in normal tissues. Therefore, inhibiting these factors in order to treat cancer may lead to severe side effects. Here, we show that activating transcription factor 5 (ATF5), highly expressed in tumors, is activated by ECM stiffness and promotes the proliferation of cancer cells, including that of pancreatic cancer cells and lung cancer cells. In addition, ATF5 suppressed the expression of early growth response 1 (EGR1), thereby accelerating cancer cell proliferation. Stiff ECMs trigger the JAK-MYC pathway which activates ATF5. JAK activation was actomyosin independent whereas MYC induction was actomyosin dependent. These results demonstrate the critical role played by ATF5 in the mechanotransduction process seen in cancers.

## Highlights

• Stiff ECMs trigger ATF5 nuclear localization in cancer cell lines and tumor tissues
• Stiff ECMs promote the proliferation of cancer cells via ATF5 activation
• ATF5 activation by stiff ECMs suppresses EGR1 expression in cancer cells
• Stiff ECMs induce the integrin-JAK-MYC and actomyosin-MYC pathways to activate ATF5

## Graphical abstract

**Figure:**
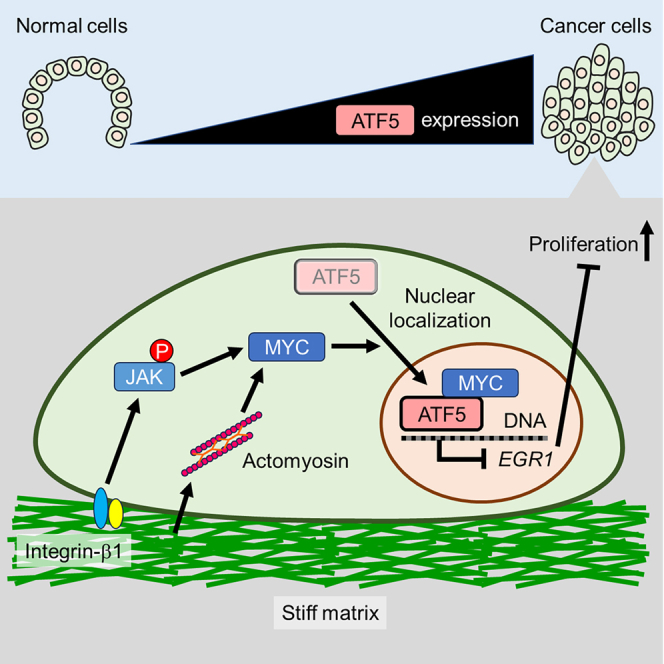

## Introduction

Cancer tissues are often stiffer than normal tissues.^1^^,^^2^ For instance, the stiffness of pancreatic cancer tissues is approximately 6 kPa, whereas that of corresponding normal tissues is approximately 2 kPa.^3^ The stiffening of a tissue is mainly caused by the stiffening of its ECM. Cancer-associated fibroblasts (CAFs), are considered to be a key group of cells associated with ECM stiffening, by virtue of secreting ECM components, such as collagens and fibronectins,^4^^,^^5^^,^^6^ contracting ECMs via contractile forces,^7^ and helping crosslink ECMs by producing crosslinkers.^8^^,^^9^ Furthermore, the stimulation of cancer cells and CAFs by stiff ECMs enhances their proliferation,^10^^,^^11^^,^^12^ migration/invasion,^7^^,^^13^^,^^14^ metastasis,^15^^,^^16^^,^^17^ and drug resistance,^18^^,^^19^^,^^20^ in various cancer cells followed by cancer progression. However, although the targeting of tissue stiffening shows potential as an effective therapeutic technique, approved therapeutic drugs aimed at tissue stiffening in cancer remain scant.

Transcription factors, which bind to DNA and regulate the expression of genes, including that of tumor-promoting and tumor-suppressing genes, are critical for cancer progression.^21^ Previous studies have identified transcription factors that are regulated by ECM stiffness. More specifically, nuclear localization of these factors initiates their function and activates cancer progression. YAP/TAZ are two of the transcriptional cofactors found to accumulate in the nuclei of cells on stiff ECMs^22^ and promote the proliferation or invasion of cancer cells.^11^^,^^23^^,^^24^ We found that NF-κB was localized in the nuclei of lung cancer cells on stiff ECMs, wherein they increased the expression of inflammatory genes involved in cancer progression.^25^ In addition, stiff ECMs trigger the nuclear localization of β-catenin and Twist1, which induces cancer progression.^26^^,^^27^ However, the known transcription factors have not fully explained the mechanism of regulating cancer progression through ECM stiffness. In particular, whether transcription factors highly expressed in cancer tissues regulate malignancy in cancer cells in an ECM stiffness-dependent manner is poorly understood.

Activating transcription factor 5 (ATF5) is a member of the ATF/cAMP response element-binding family of transcription factors.^28^ ATF5 is reported to be highly expressed in pancreatic cancer, glioma, breast cancer, lung cancer, prostate cancer, and colon cancer, among others.^29^^,^^30^^,^^31^ In addition, ATF5 has been found to be highly expressed in irradiation-tolerant lung cancer cells than in non-irradiated lung cancer cells, with such high expression being linked to enhanced proliferation and invasion of these cells.^32^ Moreover, that ATF5 contributes to cancer progression is further evidenced by its suppression of apoptosis in glioma and breast cancer cells,^33^^,^^34^ promotion of metastasis in neuroblastoma cells,^35^ and enhancement of drug resistance in pancreatic cancer cells.^30^ These findings suggest that ATF5 is a highly expressed tumor-promoting transcription factor in various cancer tissues. However, the regulatory mechanisms underlying its activation, including localization in nuclei, remain unclear.

In this study, we revealed that ECM stiffness regulates the nuclear localization of ATF5 in pancreatic and lung cancer cells. Stiff ECMs induced the nuclear localization of ATF5, whereas soft ECMs did not. Furthermore, the localization of ATF5 in the nuclei suppressed the transcription of EGR1, a protein that prevents the proliferation of cancer cells. The activation of ATF5 by a stiff ECMs is triggered by the integrin β1-JAK-MYC pathway and the actomyosin-MYC pathway, via the formation of an MYC-ATF5 complex. ATF5 is highly expressed in pancreatic cancer tissues compared with adjacent normal pancreatic tissues and localized in the nuclei of collagen-rich and stiff pancreatic cancer cells. These results indicate that ATF5 activation stimulated by stiff ECMs is critical for the progression of certain cancers.

## Results

### ATF5 is highly expressed in cancer tissues

**Figure 1:**
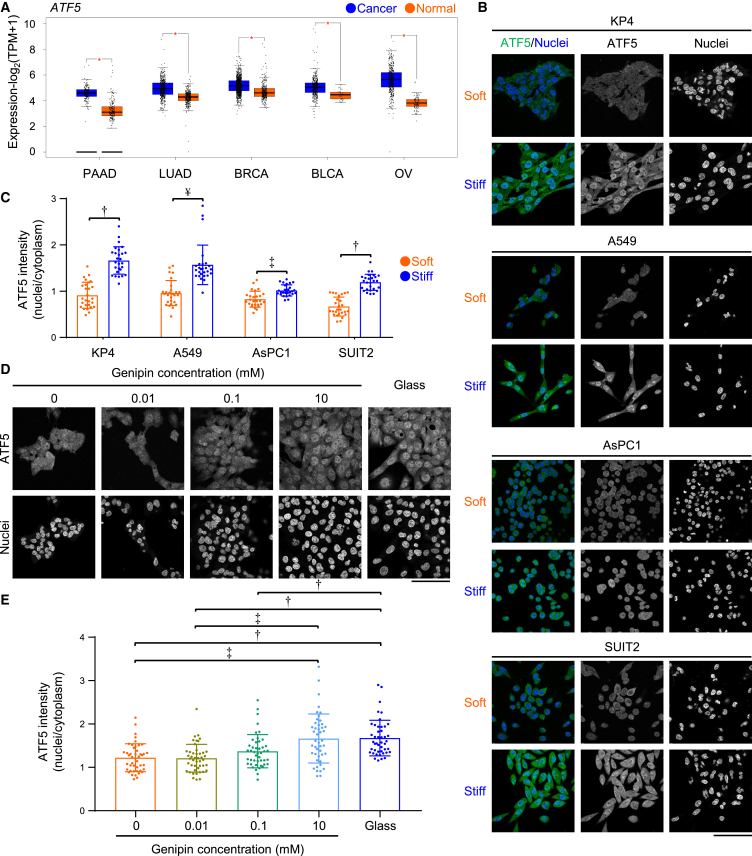
ATF5 is highly expressed in cancer tissues and localizes in the nuclei by stiff matrices (A) *ATF5* mRNA levels in pancreatic adenocarcinoma (PAAD), lung adenocarcinoma (LUAD), breast invasive carcinoma (BRCA), bladder urothelial carcinoma (BLCA), and ovarian serous cystadenocarcinoma (OV), with corresponding normal tissues. The GEPIA2 database was used with the following values for differential analysis: Log2FC-cutoff (0.58) and p value (0.05). (B) Immunofluorescent staining of ATF5 and nuclei in KP4, A549, AsPC1, and SUIT2 cells cultured on collagen gel (soft) or collagen-coated glass (stiff) substrates. (C) Quantification of the relative intensity of ATF5 in the nuclei to that in the cytoplasm, quantified from (B); *n* = 27 cells in 3 experiments. (D) Immunofluorescent staining of ATF5 and nuclei in KP4 cells on genipin-mixed collagen gel or collagen-coated glass (glass) substrates. Genipin increases the stiffness of collagen gels, as depicted by the stiffness of gels with 0, 0.01, 0.1, and 10 mM genipin being approximately 0.0292, 0.267, 1.49, and 12.5 kPa, respectively. (E) Relative intensity of ATF5 in the nuclei to that in the cytoplasm, quantified from (D); *n* = 45 cells in 3 experiments. Scale bar = 100 μm; mean with S.D. and each data point is shown; †, statistical significance (*p* < 0.05) determined via Student’s t test; ‡, statistical significance (*p* < 0.05) determined via Welch’s t-test; ¥, statistical significance (*p* < 0.05) determined via Wilcoxon rank-sum test. For multiple comparisons, we analyzed significance using the Bonferroni correction.

Previous studies have indicated that transcription factors, such as β-catenin, NF-κB, Twist1, and YAP1, are activated by stiff ECMs.^22^^,^^25^^,^^26^^,^^27^ To investigate whether certain transcription factors are highly expressed in cancer tissues, which are reported to be stiffer than corresponding normal tissues,^3^^,^^36^^,^^37^^,^^38^ we analyzed the gene expression of these factors in pancreatic, lung, breast, and bladder cancer tissues, as well as in corresponding normal tissues. We also analyzed gene expression in ovarian cancer, the stiffness of which differs depending on the subtypes.^39^ The expression levels of *CTNNB1, RELA, TWIST1*, and *YAP1* in pancreatic adenocarcinoma (PAAD) tissues were higher than those in corresponding normal tissues. However, no remarkable differences were observed between the expression levels of these factors in lung adenocarcinoma (LUAD), breast invasive carcinoma (BRCA), bladder urothelial carcinoma (BLCA), and ovarian serous cystadenocarcinoma (OV) tissues and normal tissues (Figure S1A). On the other hand, *ATF5* expression in PAAD, LUAD, BRCA, BLCA, and OV tissues was higher compared with that in corresponding normal tissues (Figure 1A). *ATF5* expression in invasive breast carcinoma subtypes (basal-like (triple negative), HER2+ non-luminal, Luminal A, and Luminal B) was also higher than that in the corresponding normal tissues (Figure S1A). These results indicate that ATF5 is highly expressed in various cancers with stiff tissues and may be a promising therapeutic target.

### Stiff extracellular matrix induce ATF5 nuclear localization in cancer cells

Next, we examined whether ATF5 is activated by stiff ECMs. Because the nuclear localization of transcription factors is critical for their activation, we examined ATF5 localization in cancer cells on stiff and soft ECMs. More ATF5 was localized in the nuclei of pancreatic cancer cells (KP4, AsPC1, and SUIT2) and lung cancer cells (A549) cultured on stiff ECMs (collagen-coated glass dishes) than that in those cultured on soft ECMs (collagen gels); (Figures 1B and 1C). Recently, we established a method for preparing collagen gels with different stiffnesses via genipin crosslinking.^40^ Using this method, we were able to culture cells on collagen gels of approximately 0.0292–12.5 kPa stiffness. Our observations confirmed that stiffer collagen gels treated with genipin triggered the nuclear localization of ATF5 in KP4 cells (Figures 1D and 1E). We also cultured KP4 cells and A549 cells on stiff (271 kPa) and soft (0.4 kPa) polyacrylamide gel substrates and found that stiffer gels induced the nuclear localization of ATF5 in these cells (Figures S1B and S1C). On the other hand, stiff ECMs did not increase ATF5 protein expression in KP4 and A549 cells (Figure S1D), suggesting that ECM stiffening promotes ATF5 activation independent of ATF5 protein expression. ATF5 has a nuclear localization sequence^41^ which is critically linked to active protein transport via importin β1 (*KPNB1*).^42^^,^^43^ We found that knockdown of *KPNB1* decreased ATF5 nuclear localization in KP4 cells (Figures S1E–S1G). Therefore, it is suggested that stiff ECMs induce the nuclear localization of ATF5 via active protein transport in cancer cells.

### Stiff matrix triggers the proliferation of cancer cells via ATF5

**Figure 2:**
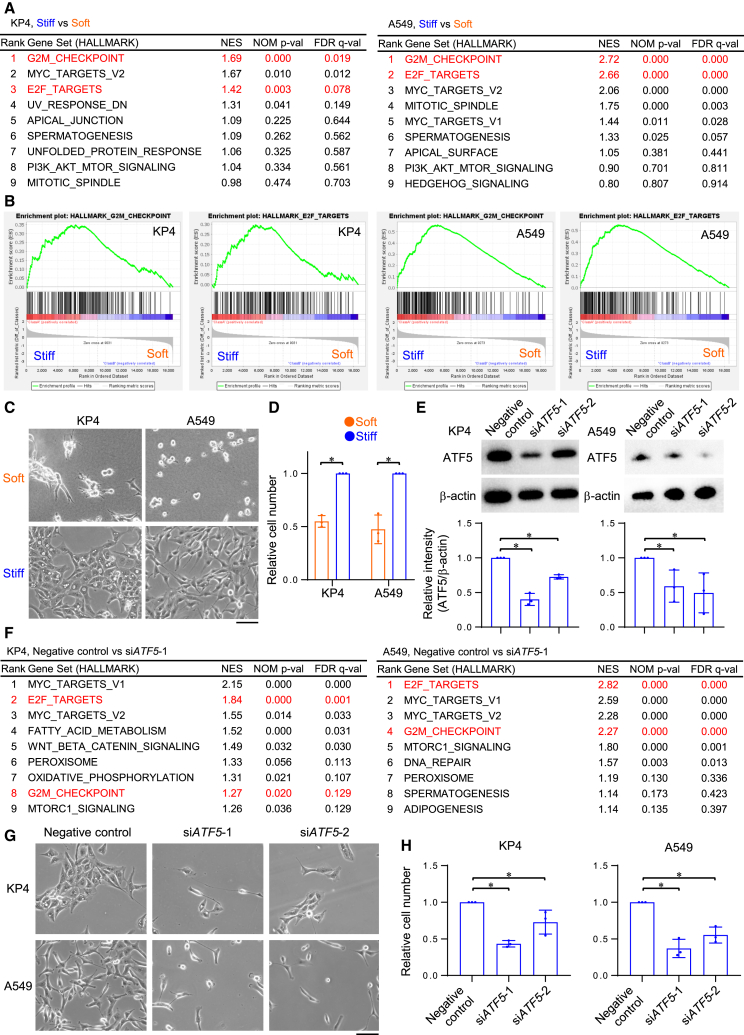
Stiff matrix triggers the proliferation of cancer cells via ATF5 (A) GSEA of upregulated genes for hallmark gene sets in KP4 or A549 cells on collagen-coated plastic (stiff) substrates compared with the cells on collagen gel (soft) substrates. NES, normalized enrichment score; NOM p-val, nominal *p* value; FDR q-val, false discovery rate q value. (B) GSEA of G2M checkpoint or E2F targets highlighted in (A). G2M checkpoint and E2F targets are associated with cell proliferation. (C) Phase-contrast images of KP4 or A549 cells on collagen gel (soft) or collagen-coated plastic (stiff) substrates. (D) Relative cell number, analyzed from (C); *n* = 3 experiments. (E) Western blot of ATF5 and β-actin in KP4 or A549 cells transfected with negative control RNA, si*ATF5*-1, or si*ATF5*-2 on collagen-coated plastic dishes. Relative intensity of ATF5 to β-actin is shown; *n* = 3 experiments. (F) GSEA of upregulated genes for hallmark gene sets in KP4 or A549 cells transfected with negative control RNA or si*ATF5*-1 on collagen-coated plastic dishes. NES, normalized enrichment score; NOM p-val, nominal *p* value. FDR q-val, false discovery rate q value. (G) Phase-contrast images of KP4 or A549 cells transfected with negative control RNA, si*ATF5*-1, or si*ATF5*-2 on collagen-coated plastic dishes. (H) Relative cell number, analyzed from (G); *n* = 3 experiments. Scale bar = 100 μm; mean with S.D. and each data point is shown; ∗, statistical significance determined with 95% confidence interval. For multiple comparisons, we analyzed significance using the Bonferroni correction.

Next, we investigated whether cellular phenotypes were being regulated by ECM stiffness and ATF5. A DNA microarray assay followed by gene set enrichment analysis (GSEA) showed that genes involved in the G2/M checkpoint (G2M CHECKPOINT) as well as genes encoding the cell cycle-related targets of E2F transcription factors (E2F TARGETS) were enriched in the KP4 and A549 cells on stiff ECMs (Figures 2A and 2B). These results, which indicated that stiff ECMs may trigger the proliferation of cancer cells, were consistent with those of previous studies.^10^^,^^11^ Indeed, the proliferation of KP4 and A549 cells on stiff ECMs was greater than that on soft ECMs (Figures 2C and 2D). On the other hand, there was no significant difference between cell survival rates on stiff and soft ECMs (Figure S2A). Next, we utilized siRNA transfection to knockdown *ATF5* in KP4 and A549 cells (Figure 2E). Similar to results seen in cells cultured on soft ECMs, *ATF5* knockdown inhibited the enrichment of genes in G2M checkpoint and E2F targets (Figures 2F and S2C) as well as cell proliferation (Figures 2G and 2H), but did not inhibit cell survival (Figure S2B). The expression of the proliferation marker, Ki67 (*MKI67*), was not significantly different between in KP4 cells on stiff and soft ECMs (Figure S2D), and not inhibited by *ATF5* knockdown in KP4 cells (Figure S2E). Therefore, ATF5 activation by stiff ECMs may accelerate cell division rate by regulating the G2M checkpoint and E2F activation. In addition, *YAP1* knockdown by siRNA transfection prevented the growth of KP4 cells on stiff ECMs (Figures S2F–S2I). GSEA showed that genes involved in YAP signaling (CORDENONSI_YAP_CONSERVED_SIGNATURE^44^) were enriched in KP4 cells on stiff ECMs (Figure S2J). These results indicate that both ATF5 and YAP1 are critical for cell proliferation in cancer cells on stiff ECMs. Collectively, these results suggested that stiff ECMs accelerate the cell cycle of KP4 and A549 cancer cells via ATF5 activation.

### ATF5 activated by stiff matrix suppresses early growth response 1 expression

**Figure 3:**
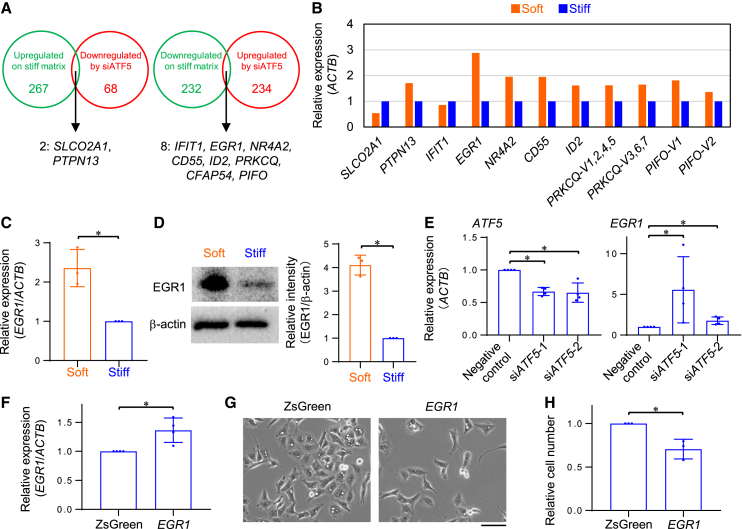
ATF5 activated by stiff matrix suppresses EGR1 expression (A) Venn diagram showing the overlap of candidate genes upregulated on stiff matrix and downregulated by si*ATF5* (left), or the overlap of candidate genes downregulated on stiff matrix and upregulated by si*ATF5* (right) in KP4 cells. (B) qPCR of candidate genes in (A) in KP4 cells on collagen gel (soft) or collagen-coated plastic (stiff) substrates. *CFAP54* was not detected by qPCR in KP4 cells. β-actin (*ACTB*) was used as an internal control; *n* = 1 experiment. We used two primer pairs to cover all transcript variants in *PRKCQ* (V1, 2, 3, 4, 5, 6, 7) and *PIFO* (V1, 2). (C) qPCR of *ATF5* and *EGR1* in KP4 cells on collagen gel (soft) or collagen-coated plastic (stiff) substrates. β-actin (*ACTB*) was used as an internal control; *n* = 3 experiments. (D) Western blot of EGR1 and β-actin in KP4 cells on collagen gel (soft) or collagen-coated plastic (stiff) substrates. Relative intensity of EGR1 to β-actin is shown; *n* = 3 experiments. (E) qPCR of *EGR1* in KP4 cells transfected with negative control RNA, si*ATF5*-1, or si*ATF5*-2 on collagen-coated plastic dishes. β-actin (*ACTB*) was used as an internal control; *n* = 4 experiments. (F) qPCR of *EGR1* in KP4 cells transfected with ZsGreen (control) or EGR1 vector for *EGR1* overexpression on collagen-coated plastic dishes. β-actin (*ACTB*) was used as an internal control; *n* = 4 experiments. (G) Phase-contrast images of KP4 cells transfected with ZsGreen or EGR1 vector for *EGR1* overexpression on collagen-coated plastic dishes. (H) Relative cell number analyzed from (G); *n* = 3 experiments. Scale bar = 100 μm; mean with S.D. and each data point is shown; ∗, statistical significance determined with 95% confidence interval. For multiple comparisons, we analyzed significance using the Bonferroni correction.

We next investigated the transcriptional targets of ATF5 that may be activated by stiff ECMs. First, we used microarray data to select the genes upregulated in KP4 cells cultured on a stiff matrix (>2) and downregulated by si*ATF5* (downregulated by si*ATF5*-1 (<0.5) and downregulated by si*ATF5*-2 (<1, due to low knockdown efficiency (Figure 2E)). As a result, we picked up *SLCO2A1* and *PTPN13*, candidate genes that may be upregulated by stiff ECMs via ATF5 activation (Figure 3A). Second, we listed the genes downregulated in the KP4 cells on stiff matrix (<0.5) and upregulated by si*ATF5* (upregulated by si*ATF5*-1 (>2) and upregulated by si*ATF5*-2 (>1)). This enabled us to identify candidate genes *IFIT1, EGR1, NR4A2, CD55, ID2, PRKCQ, CFAP54,* and *PIFO*, which may be downregulated by stiff ECMs via ATF5 activation (Figure 3A). Next, we verified these results via qPCR and found that the differences between the expression levels of *EGR1* in KP4 cells grown on stiff and soft matrices were the largest (Figure 3B). Thus, we focused on *EGR1* for further investigation. Our qPCR results indicated that *EGR1* was significantly upregulated in KP4 and A549 cells on soft ECMs compared with those grown on stiff ECMs (Figures 3C and S3A). The protein expression of EGR1 in KP4 cells on soft ECMs was also increased (Figure 3D). In addition, *EGR1* expression in KP4 and A549 cells was significantly increased due to the downregulation of *ATF5* (Figures 3E and S3B). Previous studies reported that EGR1 plays oncogenic as well as antitumor roles.^45^ Therefore, we examined whether EGR1 was critical for cell proliferation. KP4 cells overexpressing *EGR1* showed lower proliferation potential without exhibiting significant changes in cell survival on stiff ECMs (Figures 3F–3H and S3C). Furthermore, *EGR1* knockdown by siRNA transfection enhanced the proliferation of KP4 cells on soft ECMs (Figures S3D–S3G). These results indicate that although the contribution of ATF5 to the whole transcriptional profile regulated by ECM stiffness is not large (Figure 3A), stiff ECMs trigger cell proliferation by downregulating *EGR1* via ATF5 activation.

Next, we evaluated whether ATF5 and YAP1 influence each other in KP4 cells cultured on stiff or soft ECMs. We extracted the total RNA of control, *ATF5*-knock downed, and *YAP1*-knock downed KP4 cells on soft or stiff ECMs and performed qPCR of *ATF5* and *YAP1* (Figure S3H), the candidate genes regulated by both stiffness and ATF5 (Figure S3I; *SLCO2A1, PIFO, PRKCQ, ID2, CD55, NR4A2*, and *EGR1*, selected from Figure 3B), and the candidate genes upregulated by both stiff ECM and YAP overexpression (Figure S3J; *CCN2, FADD45B, AXL, TGM2, DAB2, LHFPL6, DDAH1*, and *THBS1*, selected from our microarray results and GSEA CORDENONSI_YAP_CONSERVED_SIGNATURE^44^). We successfully knocked down *ATF5* and *YAP1* in KP4 cells cultured on stiff or soft ECMs (Figure S3H). We found that the knockdown of *YAP1* did not affect *ATF5* expression, whereas *ATF5* knockdown increased *YAP1* expression in KP4 cells on both stiff and soft ECMs (Figure S3H). The genes regulated by both stiffness and ATF5 were *PIFO, PRKCQ, ID2, NR4A2*, and *EGR1*; these genes were downregulated by stiff ECMs and upregulated by *ATF5* knockdown in KP4 cells on stiff ECMs (Figure S3I). The expression of these five genes also tended to be upregulated by *ATF5* knockdown in KP4 cells on soft ECMs. In contrast, the expression of three genes (*PRKCQ, NR4A2*, and *EGR1*) was not dramatically regulated by ECM stiffness in *ATF5* knockdowned KP4 cells (Figure S3I), suggesting that KP4 cells on both stiff and soft ECMs possess ATF5 functions, although the activity of ATF5 is different. Indeed, the ATF5 signal was diffuse but was detected in KP4 cells on soft ECMs (Figures 1B, 1D, and S1B). *ATF5* knockdown prevented growth without significant changes in the survival of KP4 cells on soft ECMs (Figures S3K and S3L), and KP4 cells on stiff ECMs (Figures 2G, 2H, and S2B). The expression of these five genes was not dramatically regulated by YAP1 (Figure S3I), consistent with Figure S3H. These results suggest that ATF5 regulates the expression of *PIFO, PRKCQ, ID2, NR4A2*, and *EGR1* independent of YAP1. Furthermore, the expression of candidate genes upregulated by both stiff ECMs and YAP overexpression was enhanced by stiff ECMs in KP4 cells, consistent with the GSEA results (Figure S2J). Stiff ECMs increased YAP signature genes, but the expression of four genes (*GADD45B, AXL, DAB2*, and *THBS1*) was not enhanced by stiff ECMs in *ATF5* knockdown cells (Figure S3J). *YAP1* knockdown in KP4 cells on stiff ECMs did not decrease the expression of candidate genes upregulated by both stiff ECMs and YAP overexpression (Figure S3J), indicating that YAP1 function is compensated by other proteins. In contrast, *ATF5* knockdown increased the expression of these genes in KP4 cells (Figure S3J). These results are consistent with those shown in Figure S3H, as *ATF5* knockdown enhanced *YAP1* expression. These results indicated that ATF5 is highly activated in KP4 cells on stiff ECMs, whereas ATF5 expression in KP4 cells on both stiff and soft ECMs suppresses *YAP1* expression and subsequent gene expression regulated by YAP1 and ECM stiffness.

### Stiff matrix activates ATF5 via pJAK

**Figure 4:**
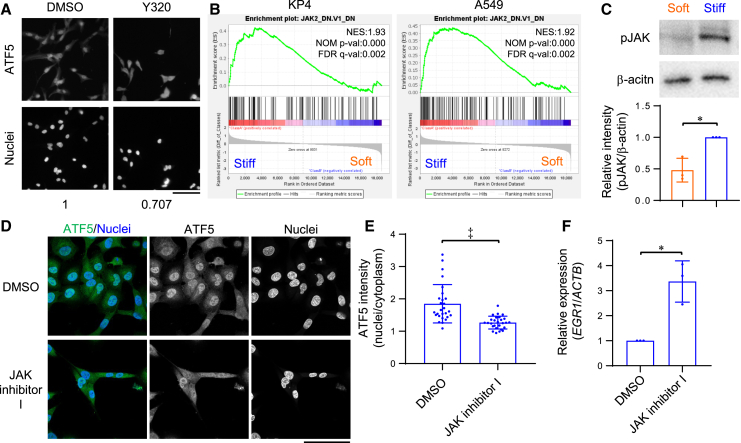
Stiff matrix activates ATF5 via pJAK (A) Immunofluorescent staining of ATF5 and nuclei in KP4 cells treated with DMSO or Y320 on collagen-coated glass plates. Y320 is reported to inhibit JAK phosphorylation. Representative images from drug screening. The value shows the relative ATF5 intensity in nuclei to cytoplasm (DMSO = 1). (B) GSEA of JAK2 DN V1 DN gene set in KP4 or A549 cells on collagen-coated plastic (stiff) substrates compared with the cells on collagen gel (soft) substrates. NES, normalized enrichment score; NOM p-val, nominal *p* value. FDR q-val, false discovery rate q value. (C) Western blot of pJAK and β-actin in KP4 cells on collagen gel (soft) or collagen-coated plastic (stiff) substrates. Relative intensity of pJAK to β-actin is shown. *n* = 3 experiments. (D) Immunofluorescent staining of ATF5 and nuclei in KP4 cells treated with DMSO or JAK inhibitor I on collagen-coated glass dishes. (E) Relative intensity of ATF5 in the nuclei to that in the cytoplasm, quantified from (D). *n* = 27 cells in 3 experiments. (F) qPCR of *EGR1* in KP4 cells treated with DMSO (the same data in Figure 6C) or JAK inhibitor I on collagen-coated plastic dishes. β-actin (*ACTB*) was used as an internal control; *n* = 3 experiments. Scale bar = 100 μm; mean with S.D. and each data point is shown; ∗, statistical significance determined with 95% confidence interval; ‡, statistical significance (*p* < 0.05) determined with Welch’s t-test.

In order to investigate the regulatory mechanisms underlying the activation of ATF5 by ECM stiffness, we performed a drug screening analysis to detect inhibitors of ATF5 activation. The top candidate was Y320 (Figures 4A and S4A), which is reported to be a potential inhibitor of JAK signaling.^46^ Next, we investigated whether stiff ECMs activate JAK signaling in cancer cells. The GSEA results showed that the genes that were downregulated following the knockdown of *JAK2* by RNAi (JAK2, DN.V1-DN) were enriched in the KP4 and A549 cells on stiff ECMs (Figure 4B). Furthermore, the levels of phosphorylated JAK in KP4 cells on stiff ECMs were significantly higher than in those on soft ECMs (Figure 4C). These results suggested that stiff ECMs may activate ATF5 by promoting JAK signaling. To verify this, we treated KP4 cells with a pan-JAK inhibitor, JAK inhibitor I, and analyzed ATF5 localization and *EGR1* expression. JAK inhibitor I inhibited the nuclear localization of ATF5 and enhanced *EGR1* expression in KP4 cells on stiff ECMs (Figures 4D–4F and S4B). In contrast, JAK inhibition in KP4 cells on soft ECMs did not significantly affect ATF5 localization (Figures S4F and S4G). In addition, specific knockdown of JAK family genes, *JAK1, JAK2, and TYK2*,^47^ significantly decreased ATF5 localization to the nucleus (Figures S4C–S4E). These results suggested that stiff ECMs trigger JAK signaling which in turn activates ATF5 leading to the downregulation of EGR1.

### Stiff matrix activates ATF5 via MYC

**Figure 5:**
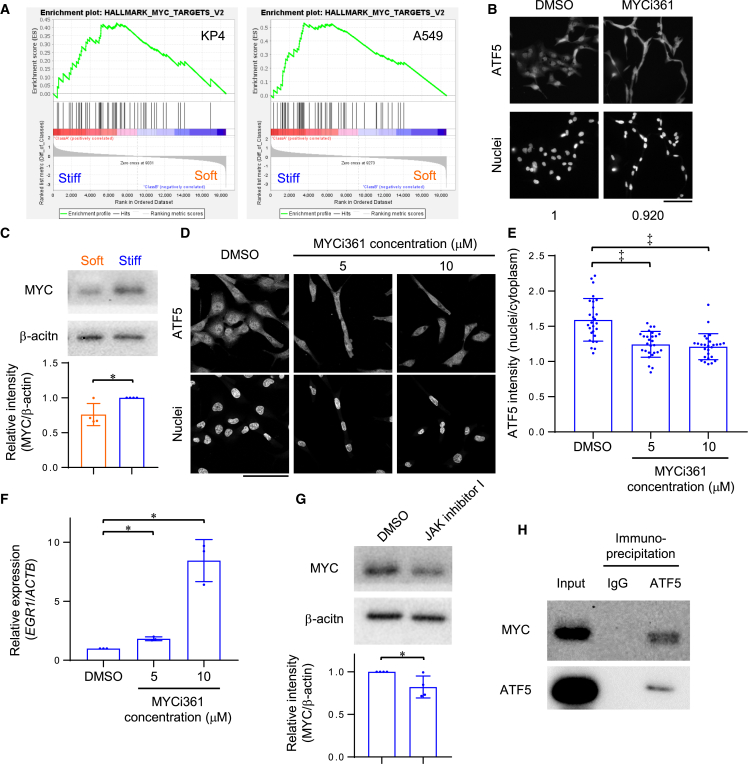
Stiff matrix activates ATF5 via MYC (A) GSEA of hallmark MYC targets V2 gene set in KP4 or A549 cells on collagen-coated plastic (stiff) substrates, compared with the cells on collagen gel (soft) substrates. (B) Immunofluorescent staining of ATF5 and nuclei in KP4 cells treated with DMSO or MYC inhibitor MYCi361 on collagen-coated glass plates. Representative images from drug screening. The value shows the relative ATF5 intensity in the nuclei to that in the cytoplasm (DMSO = 1). (C) Western blot of MYC and β-actin in KP4 cells on collagen gel (soft) or collagen-coated plastic (stiff) substrates. Relative intensity of MYC to β-actin is shown; *n* = 4 experiments. (D) Immunofluorescent staining of ATF5 and nuclei in KP4 cells treated with DMSO or MYCi361 (5 or 10 μM) on collagen-coated glass dishes. (E) Relative intensity of ATF5 in the nuclei to that in the cytoplasm, quantified from (D). *n* = 27 cells in 3 experiments. (F) qPCR of *EGR1* in KP4 cells treated with DMSO or MYCi361 (5 or 10 μM) on collagen-coated plastic dishes. β-actin (*ACTB*) was used as an internal control; *n* = 3 experiments. (G) Western blot of MYC and β-actin in KP4 cells treated with DMSO or JAK inhibitor I on collagen-coated plastic dishes. Relative intensity of MYC to β-actin is shown; *n* = 4 experiments. (H) Immunoprecipitation with control IgG or anti-ATF5 antibody followed by western blotting with anti-MYC or anti-ATF5 antibody in KP4 cells on collagen-coated plastic dishes. Input sample is shown together. Representative data of 3 experiments are shown. Scale bar = 100 μm; mean with S.D. and each data point is shown; ∗, statistical significance determined with 95% confidence interval; ‡, statistical significance (*p* < 0.05) determined via Welch’s t-test. For multiple comparisons, we analyzed significance using the Bonferroni correction.

The microarray assay followed by GSEA indicated that genes regulated by MYC (MYC TARGETS V2) in KP4 and A549 cells cultured on stiff ECMs were enriched (Figures 2A and 5A). Drug screening results also showed that the MYC inhibitor, MYCi361, reduces the nuclear localization of ATF5 (Figure 5B). Therefore, we hypothesized that MYC may play a critical role in ATF5 activation by stiff ECMs. Indeed, the expression of MYC protein in KP4 cells on stiff ECMs was higher than that on soft ECMs (Figure 5C). Treatment with MYCi361, as well as knockdown of *MYC* via siRNA transfection, significantly inhibited the nuclear localization of ATF5 in KP4 cells on stiff ECMs (Figures 5D, 5E, and S5A–S5C). *EGR1* expression was increased by treatment with MYCi361 (Figure 5F). Similar to the effect seen when culturing soft ECMs, treatment with JAK inhibitor I prevented MYC expression in cells cultured on stiff ECMs (Figure 5G), suggesting that stiff ECMs may activate the JAK-MYC-ATF5 axis in cancer cells. A previous study suggested that activated transcription factor 2 (ATF2) binds to MYC and regulates transcriptional activity.^48^ Therefore, it is possible that ATF5, which also belongs to the ATF family of transcription factors including ATF2,^49^ may bind to MYC. Immunoprecipitation analysis revealed that ATF5 binds to MYC in KP4 cells on both stiff and soft ECMs (Figures 5H and S5D), suggesting that in cancer cells on stiff ECMs, highly expressed MYC binds to ATF5 and localizes to the nuclei with ATF5. Collectively, these results indicated that stiff ECMs activate JAK signaling, leading to the formation of ATF5-MYC complexes in nuclei, thereby preventing *EGR1* transcription in cancer cells.

### Actomyosin-dependent and independent activation of ATF5

**Figure 6:**
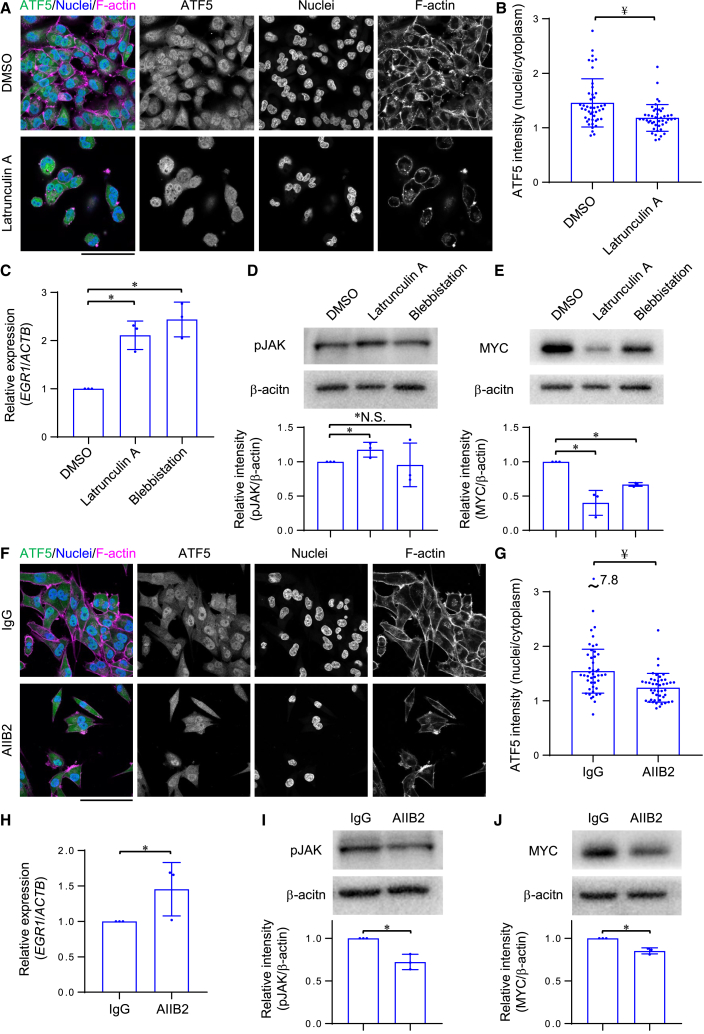
ATF5 is activated in an actomyosin-dependent or independent manner (A) Immunofluorescent staining of ATF5, nuclei, and F-actin in KP4 cells treated with DMSO or Latrunculin A (actin polymerization inhibitor) on collagen-coated glass dishes. (B) Relative intensity of ATF5 in the nuclei to that in the cytoplasm, quantified from (A). *n* = 45 cells in 3 experiments. (C) qPCR of *EGR1* in KP4 cells treated with DMSO (the same data in Figure 4F), Latrunculin A, or Blebbistatin (myosin II inhibitor) on collagen-coated plastic dishes. β-actin (*ACTB*) was used as an internal control; *n* = 3 experiments. (D) Western blot of pJAK and β-actin in KP4 cells treated with DMSO, Latrunculin A, or Blebbistatin on collagen-coated plastic dishes. Relative intensity of pJAK to β-actin is shown; *n* = 3 experiments. (E) Western blot of MYC and β-actin in KP4 cells treated with DMSO, Latrunculin A, or Blebbistatin on collagen-coated plastic dishes. Relative intensity of MYC to β-actin is shown; *n* = 3 experiments. (F) Immunofluorescent staining of ATF5, nuclei, and F-actin in KP4 cells treated with control IgG or AIIB2 (integrin β1 blocking antibody) on collagen-coated glass dishes. (G) Relative intensity of ATF5 in the nuclei to that in the cytoplasm, quantified from (F); *n* = 45 cells in 3 experiments. (H) qPCR of *EGR1* in KP4 cells treated with control IgG or AIIB2 on collagen-coated plastic dishes. β-actin (*ACTB*) was used as an internal control; *n* = 3 experiments. (I) Western blot of pJAK and β-actin in KP4 cells treated with control IgG or AIIB2 on collagen-coated plastic dishes. Relative intensity of pJAK to β-actin is shown; *n* = 3 experiments. (J) Western blot of MYC and β-actin in KP4 cells treated with control IgG or AIIB2 on collagen-coated plastic dishes. Relative intensity of MYC to β-actin is shown; *n* = 3 experiments. Scale bar = 100 μm; mean with S.D. and each data point is shown; ∗, statistical significance determined with 95% confidence interval; ¥, statistical significance (*p* < 0.05) determined via Wilcoxon rank-sum test. For multiple comparisons, we analyzed significance using the Bonferroni correction.

Previous studies have suggested that actomyosin and adhesion proteins play an important role in the cellular response to ECM stiffness.^11^^,^^25^^,^^37^^,^^50^ Therefore, we treated cancer cells with inhibitors of actomyosin and adhesion molecules, and then analyzed ATF5 localization, *EGR1* expression, JAK phosphorylation, and MYC expression. Actomyosin consists of F-actin and myosin II which generate contractile forces.^51^ The F-actin inhibitor, Latrunculin A, and the myosin II inhibitor, blebbistatin, decreased nuclear localization of ATF5 (Figures 6A, 6B, S6A, and S6B) and increased *EGR1* expression (Figure 6C). On the other hand, Y27632, which decreases actomyosin contraction by inhibiting the Rho-ROCK pathway,^52^ did not significantly inhibit the nuclear localization of ATF5 (Figures S6C and S6D), suggesting that ATF5 activation was not dependent on Rho-ROCK signaling. Treatment with Latrunculin A, or blebbistatin, did not significantly decrease the level of phosphorylated JAK (Figure 6D), whereas they significantly suppressed MYC expression (Figure 6E). These results indicated that actomyosin activates ATF5 via MYC expression in a manner independent of JAK signaling.

Next, we examined the contribution of adhesion molecules, which play a critical role in cell-ECM adhesion. The inhibition of integrin β1, which is a key adhesion molecule that binds cells to the ECM and thereby contributes to stiffness sensing,^53^ using the inhibitory monoclonal antibody, AIIB2,^54^ significantly suppressed the nuclear localization of ATF5 in KP4 cells on stiff ECMs (Figures 6F and 6G). Moreover, AIIB2 elevated *EGR1* expression (Figure 6H), decreased the level of phosphorylated JAK (Figure 6I), and suppressed MYC expression (Figure 6J), suggesting that cell-ECM adhesion via integrins was critical for activating JAK signaling to induce MYC expression, which is critical for the activation of ATF5 by stiff ECMs. On the other hand, the inhibition of focal adhesion kinase (FAK), which is reported to transduce mechanical stimuli, such as ECM stiffness, into biochemical signaling,^55^ was insufficient to prevent the nuclear localization of ATF5 in KP4 cells on stiff ECMs (Figures S6E and S6F). This result indicated that ATF5 activation by ECM stiffness is regulated by cell-ECM adhesion, and not via FAK signaling in cancer cells.

We also investigated whether cell contractility and F-actin are regulated by ECM stiffness and whether they are critical for ATF5 nuclear localization. We performed immunofluorescence staining of the nuclei, di-phosphorylated MRLC (PP-MRLC; a marker of cell contractility by actomyosin^52^), and F-actin in KP4 cells (Figure S6G). PP-MRLC intensity was increased by stiff ECMs in KP4 cells and by Calyculin A (a phosphatase inhibitor) in KP4 cells cultured on soft ECMs, whereas it was decreased by Y27632 in KP4 cells cultured on stiff ECMs. F-actin was increased by stiff ECMs in KP4 cells and decreased by Latrunculin A and Blebbistatin in KP4 cells on stiff ECMs. Because ATF5 nuclear localization was prevented by Latrunculin A and Blebbistatin in KP4 cells on stiff ECMs whereas it was not significantly prevented by Y27632 in KP4 cells on stiff ECMs nor elevated by Calycilin A in KP4 cells on soft ECMs (Figures 6A, 6B, S6A–S6D, S6H, and S6I), it is indicated that F-actin is critical for ATF5 nuclear localization whereas cell contractility by PP-MRLC is not. Furthermore, AIIB2 treatment of KP4 cells on stiff ECMs did not remarkably affect PP-MRLC and F-actin (Figure S6G), suggesting that integrin β1 promotes ATF5 nuclear localization independent of actomyosin. In addition, Rac1 (*RAC1*) knockdown in KP4 cells on stiff ECMs did not remarkably affect the level of pJAK (Figures S6J and S6K), suggesting that the ROCK-independent Rac1 pathway, regulating actomyosin^56^ is not critical for JAK signaling.

### ATF5 activation triggered by the stiff matrix is critical for tumor growth *in vivo*

**Figure 7:**
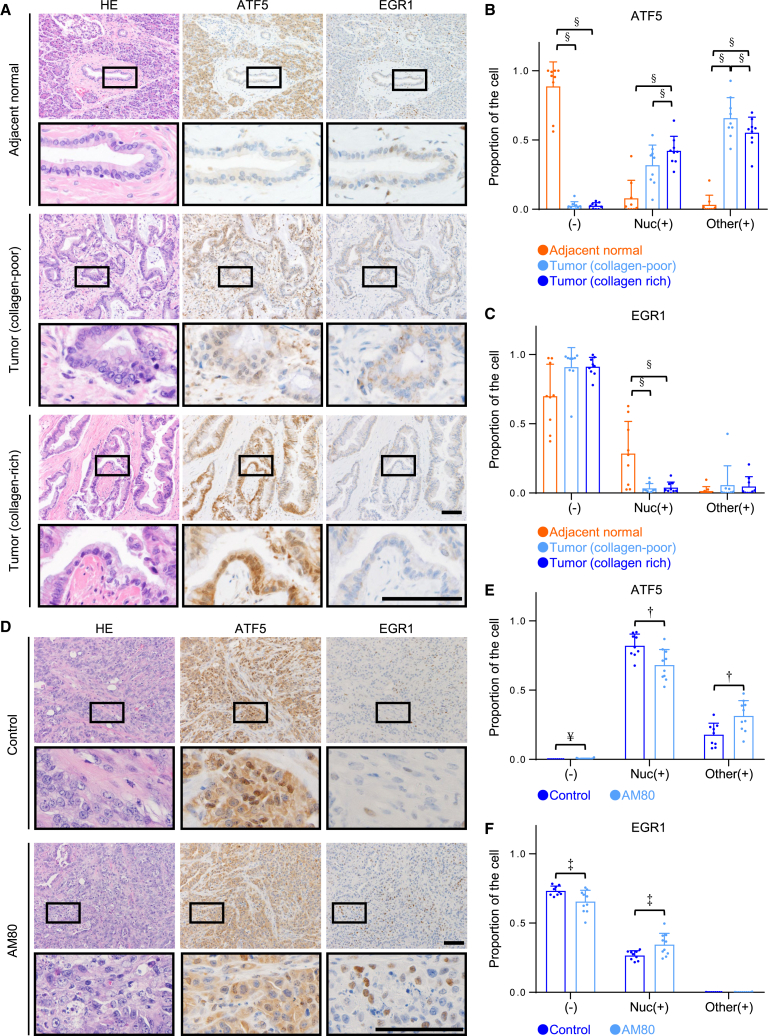
ATF5 is highly localized in the nuclei of human and mouse pancreatic cancer cells in stiff tumors (A) Representative staining images of hematoxylin and eosin (HE) and immunohistochemistry for ATF5 and EGR1 in adjacent normal, and collagen-poor and collagen-rich tumor regions of human pancreatic ductal adenocarcinoma. The areas of black rectangles are magnified. (B) Proportions of cells negative for ATF5 (−), nuclear positive for ATF5 (Nuc(+)), and nuclear negative and cytoplasm-positive for ATF5 (Other(+)) in human pancreatic cancer tissues (*n* = 9 patients) were examined via immunohistochemistry, followed by quantification. (C) Proportions of cells negative for EGR1 (−), nuclear-positive for EGR1 (Nuc(+)), and nuclear-negative and cytoplasm-positive for EGR1 (Other(+)) in human pancreatic cancer tissues (*n* = 9 patients) were examined by immunohistochemistry, followed by quantification. (D) Representative staining images of hematoxylin and eosin (HE) and immunohistochemistry for ATF5 and EGR1 in control pancreatic tumors and those treated with AM80. The areas of black rectangles are magnified. Note that AM80 is reported to reduce tumor stiffness by inducing changes in the phenotype of CAFs. (E) Proportions of cells negative for ATF5 (−), nuclear positive for ATF5 (Nuc(+)), and nuclear negative and cytoplasm-positive for ATF5 (Other(+)) in tumor tissues obtained from control mice (*n* = 9) and those administered AM80 (*n* = 10) were examined via immunohistochemistry followed by quantification. (F) Proportions of cells negative for EGR1 (−), nuclear positive for EGR1 (Nuc(+)), and nuclear negative and cytoplasm-positive for EGR1 (Other(+)) on tumor tissues obtained from control mice (*n* = 9) and those administered AM80 (*n* = 10) were examined via immunohistochemistry followed by quantification. Scale bar = 100 μm; mean with S.D. and each data point is shown; †, statistical significance (*p* < 0.05) determined via Student’s t test; ‡, statistical significance (*p* < 0.05) determined via Welch’s t-test; ¥, statistical significance (*p* < 0.05) determined via Wilcoxon rank-sum test; §, statistical significance (*p* < 0.05) determined via paired t-test. For multiple comparisons, we analyzed significance using the Bonferroni correction.

Finally, we evaluated whether ATF5 is highly expressed and localized in the cellular nuclei of human pancreatic tissues obtained from patients with pancreatic ductal adenocarcinoma in our institution. We examined the level and localization of ATF5, as well as those of its target, EGR1, in pancreatic tissues via immunohistochemistry, and found that they were differentially expressed both in the cytoplasm and the nuclei of the epithelial cells of tumors and adjacent normal tissues (Figure S7A). We confirmed that ATF5 levels in pancreatic cancer tissues were significantly higher than those in corresponding adjacent normal tissues (Figures 7A and 7B). Furthermore, the nuclear localization of ATF5 was significantly greater in collagen-rich tumor regions, which is reported to exhibit stiffer ECMs,^57^^,^^58^ than collagen-poor tumor tissues which represent softer regions (Figures 7A and 7B). The Human Protein Atlas database also shows that ATF5 is expressed and localized to the nuclus in several cancers, including lung, breast, and prostate (https://www.proteinatlas.org/ENSG00000169136-ATF5/pathology). By contrast, the nuclear EGR1 level was significantly lower in tumor tissues (Figures 7A and 7C).

We further analyzed the expression of ATF5 and EGR1 in mouse pancreatic cancer tissues treated with or without AM80, which reduces tissue stiffness by increasing the number of meflin-positive cancer-restraining CAFs.^9^ AM80 treatment significantly reduced the nuclear localization of ATF5 and induced EGR1 expression in the nuclei of tumor tissues (Figures 7D–7F). Furthermore, we found that the knockdown of endogenous *ATF5* in KP4 cells significantly affected their growth, both in culture and in a subcutaneous transplantation mouse model, compared with that in control KP4 cells (Figures S7B–S7G). This result is consistent with our previous report that ATF5 overexpression increases tumor growth in A549 lung cancer cells in nude mice.^32^ Collectively, these analyses of human pancreatic cancer tissues and the xenograft tumor mouse model indicated that stiff ECMs triggered ATF5 nuclear localization, prevented EGR1 expression in pancreatic cancer cells, and enhanced tumor growth, thereby substantiating the results of *in vitro* cell culture experiments.

## Discussion

In this study, we found that ATF5 was localized in the nuclei of cancer cells cultured on stiff ECMs. Previous studies have reported that transcription factors, such as YAP/TAZ, NF-κB, β-catenin, and Twist1, were also localized in the nuclei of cancer cells on stiff substrates.^22^^,^^25^^,^^26^^,^^27^ In this study, we were able to confirm the findings of previous studies^29^^,^^30^^,^^31^ which indicated that ATF5 was highly expressed in cancer tissues, such as those of pancreas, lung, breast, and bladder cancers, which are stiffer than the corresponding normal tissues.^3^^,^^36^^,^^37^^,^^38^ Furthermore, we found that ATF5 was highly localized in the nuclei of collagen-rich pancreatic cancer tissues than in those of collagen-poor pancreatic cancer tissues. Collagen accumulation is considered to be a key factor involved in tissue stiffening,^1^^,^^58^ suggesting that ATF5 is highly expressed in cancer tissues and activated by stiff ECMs. Indeed, in mouse pancreatic cancer tissues, ATF5 is more highly localized to the nucleus in stiff than in soft tissues. This study elucidates the regulatory mechanism underlying the nuclear localization of ATF5 induced by ECM stiffness. Although previous reports have indicated that ATF5 regulation is dependent on its transcription or proteosomal degradation,^31^^,^^59^ here, we reveal that the nuclear localization of ATF5, which is critical for its functioning, is regulated by ECM stiffness in a manner independent of its protein expression. Thus, our findings have led us to propose a regulatory mechanism that underlies ATF5 activity in cancer cells.

We have previously reported that ATF5 triggers proliferation, invasion, and irradiation resistance in lung cancer cells.^32^ Other studies have also suggested that ATF5 may prevent apoptosis and enhance invasion in multiple cancer cells.^60^^,^^61^ These reports have indicated that ATF5 may act as a cancer-promoting factor in a broad range of cancer cells. Similar to those studies, here we show that stiff ECMs cause ATF5 to localize to the nuclei, from where it induces the proliferation of pancreatic and lung cancer cells. As reported previously, ECM stiffening is a factor that plays a critical role in the progression of various cancers.^1^ Thus, our findings suggest that stiff ECMs activate ATF5 in cancer tissues and promote cancer progression by driving cell proliferation.

We also revealed that ATF5 in cells on stiff ECMs decreases *EGR1* expression at the transcription stage. We previously showed that transcriptional activity of the cAMP response element (CRE), downstream, is suppressed by ATF5 in lung cancer cells.^32^ The *EGR1* promoter contains multiple CRE regions,^45^ indicating that ATF5 suppresses *EGR1* expression in cancer cells on stiff ECMs by binding to CRE. Previous studies showed that EGR1 plays bidirectional roles in cancer progression. To suppress cancer progression, EGR1 enhances the gene expression of apoptosis-inducers, suppressors of metastasis and invasion, and anti-angiogenesis factors.^45^ In addition, EGR1 is also essential for inducing cell death in pancreatic cancer cells via drug treatment.^62^ On the other hand, in glioma and breast cancer cells, ATF5 promotes *EGR1* expression via its association with p300 and binding to ATF5 response elements, which contribute to the proliferation and survival of cancer cells.^34^ Although the mechanism underlying the role played by EGR1 in tumor-promotion or tumor-suppression is not well understood, we suggest that ECM stiffness is a key factor in EGR1-dependent cancer suppression as stiff ECMs promote the proliferation of cancer cells by activating ATF5 followed by EGR1 suppression.

Our results indicate whether ATF5 and YAP1 influence each other in KP4 cells cultured on stiff or soft ECMs. We found that *ATF5* knockdown in KP4 cells, on both stiff and soft ECMs, enhanced the expression of *YAP1* and the genes upregulated by stiff ECM and YAP overexpression. A previous study reported that CRE-binding protein (CREB) binds to *YAP1* promoter and increases *YAP1* transcription in the liver.^63^ ATF5 is composed of an amphipathic leucine zipper that mediates hetero- and homodimerization,^31^ as well as CREB; therefore, ATF5 may bind to CREB and inhibit its transcriptional activation. Indeed, we have previously shown that *ATF5* knockdown enhances CRE transcriptional activity,^32^ promoted by CREB.^64^ Thus, ATF5 may suppress *YAP1* transcription by binding to CREB.

We also found that *ATF5* knockdown enhanced the expression of genes upregulated by stiff ECMs and YAP overexpression, whereas *YAP1* knockdown did not decrease their expression in KP4 cells. Previous studies reported that these genes are dependent on the TEA domain (TEAD) family of proteins, which are co-activators of YAP1.^65^^,^^66^ In addition to YAP1, TAZ is a co-activator of TEAD; therefore, TAZ may compensate for YAP1 function. Indeed, *CCN2* expression was not decreased by *YAP1* knockdown but increased by *YAP1* overexpression in liver cancer cells,^67^ consistent with our results, showing that *ATF5* knockdown enhanced the expression of *YAP1* and genes upregulated by both stiff ECM and YAP overexpression. Therefore, it is indicated that *ATF5* knockdown promotes the expression of *YAP1*, and as a result, increases the target genes of the YAP/TAZ-TEAD transcription factor complex.

We found that *YAP1* knockdown decreased the growth of KP4 cells on stiff ECMs, suggesting that YAP1 suppressed the growth of pancreatic cancer cells independently of TEAD family proteins. Previous research showed that YAP1 binds to KLF Transcription Factor 5 (KLF5) dependent on the two WW domains of YAP1 and enhances the growth of breast cancer cells.^68^ Five of nine YAP1 isoforms contain two WW domains, whereas TAZ has only one,^69^ suggesting that the binding potential of TAZ to KLF5 is lower than that of YAP1. KLF5 also contributes to pancreatic cancer cell growth.^70^ Collectively, these results suggest that YAP1 binds to KLF5 and promotes the growth of pancreatic cancer cells, independent of TEAD and TAZ, and also indicate that ATF5 enhances the growth of pancreatic cancer cells by suppressing *EGR1* expression. At the same time, ATF5 prevents the growth of pancreatic cancer cells by promoting *YAP1* expression. Therefore, the simultaneous inhibition of ATF5 and YAP1 may be an effective therapy for pancreatic cancer.

We also identified the integrin β1-JAK-MYC-ATF5 and actomyosin-MYC-ATF5 signaling pathways as stiffness-dependent pathways that promote cancer growth. Previous studies have indicated that JAK signaling followed by MYC activation may contribute to the progression of various cancers.^71^^,^^72^ In addition, MYC expression in glioma cells is induced by integrin α5β1-dependent JAK, and STAT3 activation, which follows, which in turn are regulated by stiff ECMs.^73^ Furthermore, stiff ECMs lead to integrin β1-dependent β-catenin activation, which triggers *MYC* expression in breast cancer cells.^74^ Therefore, it is possible that other transcription factors, such as STAT3 and β-catenin, are also involved in the signaling pathways regulated by ECM stiffness. We also found that F-actin inhibition prevented ATF5 localization; however, the inhibition of the Rho-ROCK pathway and FAK did not affect ATF5 localization. These results suggested that other pathways or molecules regulating actomyosin (e.g., myosin light-chain kinase or zipper interacting protein kinase^75^) or cell-ECM adhesion (e.g., talin^76^) may play a critical role in the activation of ATF5 in cancer cells on stiff ECMs. Especially, we revealed that F-actin is necessary for ATF5-dependent responses in pancreatic cancer cells, whereas the phosphorylation of the myosin regulatory right chain is not critical. We also showed that Blebbistatin, an inhibitor of myosin motor activity, inhibited ATF5 activity. Myosin phosphorylation was not critical for ATF5 activity; however, the F-actin inhibitor and Blebbistatin, which reduced F-actin intensity, inhibited ATF5 activity. These results suggested that F-actin stability is critical for ATF5 activation. Our previous study showed that F-actin stability, not myosin phosphorylation, promotes the localization of MRLC to the nucleus as a transcription factor.^75^ Therefore, the stabilization of F-actin may be critical for the activation of transcription factors, including ATF5. In addition, we showed that F-actin is important for MYC protein expression in cancer cells on stiff ECMs. Therefore, we identified the F-actin-MYC-ATF5 pathway in cancer cells on cultured on stiff ECMs.

We propose that ATF5 shows potential as a potent therapeutic target in tumors, with particular reference to stiff cancers, such as pancreatic and lung cancers.^1^ We recently showed that AM80 drug treatment increased the expression of meflin, a marker of tumor-restraining CAFs,^77^ decreased the stiffness of cancer tissues, and enhanced the effects of chemotherapy in a mouse pancreatic cancer model.^9^ Therefore, targeting tissue stiffness of tumors may present a promising approach toward improving cancer treatment. Previous studies have suggested that interfering with ATF5 via dominant negative ATF5 may be considered as a therapeutic strategy.^31^^,^^61^ However, although specific small molecules capable of inhibiting ATF5 activity are yet to be reported, these may act as potent drugs that may be utilized to counter stiff cancers, including refractory pancreatic cancers.

In summary, here, we reveal mechanotransduction pathways involving ATF5 in cancer cells. Stiff ECMs appear to elevate the level of JAK phosphorylation and increase MYC expression dependent on integrin β1. Stiff ECMs also increased MYC expression regulated by actomyosin. ATF5 binds to MYC and localizes in the nuclei, where it suppresses *EGR1* expression. These pathways are critical for the proliferation of cancer cells on stiff ECMs. Therefore, ECM stiffening and ATF5 show potential as potent therapeutic targets in stiff tumors, such as pancreatic, lung, breast, and bladder cancers.

### Limitations of the study

In this study, we revealed that ATF5 activated by stiff ECMs promoted the proliferation of cancer cells via the transcriptional suppression of *EGR1*. We showed that ATF5 was activated by stiff ECMs in pancreatic and lung cancer cells. For a detailed analysis, we used pancreatic cancer cells. However, whether ATF5 is activated by ECM stiffness in normal cells has not been investigated. Moreover, although ATF5 expression in normal tissues is lower than that in cancer tissues, there may be a function of ATF5 in corresponding normal tissues, such as the epithelial tissues of the pancreas and lungs. Our findings are limited to the context of specific cancer cells; therefore, whether ATF5 is activated by stiff ECMs or is critical for proliferation in other cells, including normal and cancer cells, should be elucidated in the future.

## Resource availability

### Lead contact

Further information and requests for resources and reagents should be directed to and will be fulfilled by the lead contact, Hisashi Haga (haga@sci.hokudai.ac.jp).

### Materials availability

All unique materials in this study will be available from the lead contact with a completed materials transfer agreement.

### Data and code availability

Microarray data (GSE252565) in this study are deposited and will be publicly available as of the date of publication in the Gene Expression Omnibus (GEO) at the National Center for Biotechnology Information (NCBI). This article does not report the original code. Any additional information required to reanalyze the data reported in this article is available from the lead contact upon request.

## Acknowledgments

The authors thank Hatsumi Sano for technical support, ACEL, Inc for drug screening analysis, and all the present and former members of the Haga laboratory at Hokkaido University for helpful support. This work was supported by Advanced Research and Development Programs for Medical Innovation by Japan Agency for Medical Research and Development (AMED) Grant Number JP16gm0810007 to H.H., 10.13039/100009619AMED under Grant Number JP17gm0810011 to H.H., JP22ym0126814 to H.H., JP19gm1210009 to A.E.; 10.13039/501100001691JSPS KAKENHI Grant Numbers JP17K07150 to H.H., JP21K07142 to H.H., JP18K15232 to S.I., JP21K07141 to S.I., JP23KK0143 to S.I., JP24H01917 to S.I., JP24K10302 to S.I.; the cancer research grant of 10.13039/100019356SGH Foundation to S.I.; Grants-in-Aid for Regional R&D Proposal-Based Program from Northern Advancement Center for Science & Technology of Hokkaido Japan to S.I.; the research grant of 10.13039/501100007263Astellas Foundation for Research on Metabolic Disorders to S.I.; the research grant of The 10.13039/100008732Uehara Memorial Foundation to S.I.; the research grant of The 10.13039/501100008673Yasuda Medical Foundation to S.I.; the research grant of The 10.13039/100007591Akiyama Life Science Foundation to S.I.; Extramural Collaborative Research Grant of 10.13039/100018501Cancer Research Institute, Kanazawa University to S.I.; Next Generation Leader Training Program of Hokkaido University to S.I.; Next Generation Life Science Collaborative Research Projects of Hokkaido University to S.I.; Support for collaboration research of young cancer scientists by scientific support programs for cancer research grant-in-aid for scientific research on innovative areas to A.N. and A.I.N.; periodic donations from M.D. Mariko Takamura to H.H.; A.N. was supported by JSPS Research Fellowship for Young Scientists (Grant Number 17J06406).

## Author contributions

Conceptualization, S.I. and H.H.; methodology, S.I., A.E., S.T., and H.H.; validation, S.I., and H.H.; formal analysis, S.I. and S.T.; investigation, S.I., A.E., A.S., and T.I.; resources, S.I., A.E., A.S., T.I., A.N., M.Y., and H.H.; writing-original draft, S.I., A.E., T.I., and H.H.; writing-review and editing, S.I., A.E., A.S., T.I., S.T., N.K., A.N., A.I.N., M.Y., T.T., and H.H.; visualization, S.I. and H.H.; supervision, S.I., A.E., and H.H.; project administration, S.I. and H.H.; funding acquisition, S.I., A.E., N.K., A.N., A.I.N., and H.H.

## Declaration of interests

The authors declare no competing interests.

## STAR★Methods

### Key resources table

**Table:** 

REAGENT or RESOURCE	SOURCE	IDENTIFIER
**Antibodies**		

IgG rat from serum	Sigma-Aldrich	Cat# I8015; RRID: AB_1163629
Normal Rat IgG, Whole Molecule, Purified	Wako	Cat# 147-09521
AIIB2-c	Developmental Studies Hybridoma Bank at the University of Iowa	RRID: AB_528306
Normal Rabbit IgG, Whole Molecule, Purified	Wako	Cat# 148–09551; RRID: AB_2920644
Anti-ATF5, Rabbit-Mono(SD2099)	Novus Biologicals	Cat# NBP2-67767
Goat anti-Rabbit IgG(H + L), Superclonal™ Recombinant Secondary Antibody, Alexa Fluor™ 488	Invitrogen	Cat# A27034
Anti-beta actin antibody (AC-15)	Abcam	Cat# ab6276; RRID: AB_2223210
Phospho-Myosin Light Chain 2 (Thr18/Ser19) Antibody	Cell Signaling Technology	Cat# 3674S; RRID: AB_2147464
EGR1(15F7)Rabitt mAb	Cell Signaling Technology	Cat# 4153S; RRID: AB_2097038
Phospho-Jak1 (Tyr1034/1035) Antibody	Cell Signaling Technology	Cat# 3331S; RRID: AB_2265057
Anti-*c*-Myc-ChIP Grade	Abcam	Cat# ab32072; RRID: AB_731658
Myc Antibody (9E10)	Santa Cruz	sc-40; RRID: AB_627268
Anti-mouse IgG, HRP-linked Antibody	Cell Signaling Technology	Cat# 7076S; RRID: AB_330924
Anti-rabbit IgG, HRP-linked Antibody	Cell Signaling Technology	Cat# 7074S; RRID: AB_2099233
ImmPRESS reagent HRP goat anti-rabbit IgG	Vector Laboratories	Cat# MP-7451; RRID: AB_2631198

**Biological samples**		

Tissue samples of human pancreatic ductal adenocarcinoma	Nagoya University Graduate School of Medicine	approval number: 2017-0127-04

**Chemicals, peptides, and recombinant proteins**		

Dulbecco’s Modified Eagle Medium	Sigma-Aldrich	Cat# D6046
Dulbecco’s Modified Eagle Medium	Nacalai Tesque	Cat# 08456-65
RPMI 1640 Medium	Gibco	Cat# 11875-119
Dulbecco’s Modified Eagle Medium	Sigma-Aldrich	Cat# D5796
Fetal Bovine Serum	Sigma-Aldrich	Cat# 172012-500ML
Antibiotic Antimycotic Solution	Sigma-Aldrich	Cat# A5955-100ML
KOD Plus *Neo*	TOYOBO	Cat# KOD-401
NucleoSpin Gel and PCR Clean-up	TAKARA	Cat# U0609B
In-Fusion HD Cloning Kit	Takara	Cat# Z9649N
NheI-HF	New England Biolabs	Cat# R3131S
EcoRI-HF	New England Biolabs	Cat# R3101S
TransIT-X2® Dynamic Delivery System	Takara	Cat# V6103
G-418 Sulfate	Promega	Cat# V7983
Puromycin	Sigma-Aldrich	Cat# P9620
Dimethyl Sulfoxide (DMSO)	Wako	Cat# 046-21981
JAK Inhibitor I	Sigma-Aldrich	Cat# 420099
MYCi361 5mg	TargetMol	Cat# T12132
Latrunculin A	Wako	Cat# 125-04363
Blebbistatin(+/−) 5mg	Enzo Life Sciences	Cat# BML-EI315-0005
Y27632	Sigma-Aldrich	Cat# Y0503
PF-562271	Merck	Cat# PZ0387
Calyculin A	Sigma-Aldrich	Cat# 208851
Cellmatrix Type I-C	Nitta Gelatin	NA
Atelocollagen Acidic Solution	KOKEN	Cat# IPC-50
Cellmatrix Type I-P	Nitta Gelatin	NA
Genipin	Wako	Cat# 078-03021
Hoechst 33342, Trihydrochloride, Trihydrate	Invitrogen	Cat# H1399
Alexa Fluor™ 546 Phalloidin	Invitrogen	Cat# A22283
Collagenase	Wako	Cat# 034-22363
Can Get Signal Solution 1 & 2	TOYOBO	Cat# NKB-101
Lipofectamine RNAiMAX Transfection Reagent	Invitrogen	Cat# 13778150
*in vitro* Transcription T7 Kit	TAKARA	Cat# 6140
Klenow Fragment (3'→5' exo-)	New England Biolabs	Cat# M0212L
TriPure Isolation Reagent	Roche	Cat# 11667165001
FastGene RNA Basic Kit	NIPPON Genetics	Cat# FG-80250
Fast Gene RNA Premium kit	NIPPON Genetics	Cat# FG-81050
Capturem IP & Co-IP kit	Clontech	Cat# 635721
Trypsin-EDTA (0.25%), phenol red	Gibco	Cat# 25200-072
Trypan Blue Stain (0.4%) for use with the Countess™ Automated Cell Counter	Invitrogen	Cat# T10282
AM80	Tocris	Cat# 3507
Liquid DAB+ Substrate Chromogen System	Dako	Cat# K3468
Entellan mounting media	Merck	Cat# 100869

**Critical commercial assays**		

MycoAlert Assay Control Set	Lonza Bioscience	Cat# LT07-518
Immobilon Western Chemiluminescent HRP substrate	Millipore	Cat# WBKLS0500
ReverTra Ace qPCR RT Master Mix	TOYOBO	Cat# FSQ-201
KAPA SYBR Fast qPCR kit	NIPPON Genetics	Cat# KK4602

**Deposited data**		

Microarray raw data	This paper	GEO: GSE252565

**Experimental models: Cell lines**		

KP4	Riken Cell Bank	Cat# RCB1005; RRID: CVCL_1338
A549	American Type Culture Collection	Cat# CCL-185; RRID: CVCL_0023
SUIT2	JCRB cell bank	Cat# JCRB1094; RRID: CVCL_3172
AsPC1	American Type Culture Collection	Cat# CRL-1682; RRID: CVCL_0152
mT5	provided by David Tuveson (Cold Spring Harbor Laboratory)	NA
HEK293	Riken Cell Bank	Cat# RCB1637; RRID: CVCL_0045

**Experimental models: Organisms/strains**		

8-weeks-old C57BL/6 female mice	The Jackson Laboratory	RRID:IMSR_JAX:000664
7-weeks-old Nude(BALB/c-nu) female mice	The Jackson Laboratory	RRID:IMSR_JAX:002019

**Oligonucleotides**		

See method details		

**Recombinant DNA**		

pIRES2-ZsGreen1 Vector	Clontech	Cat# 632478
pIRES2-EGR1-ZsGreen1	This paper	NA
GIPZ Non-silencing Lentiviral shRNA Control	Dharmacon	Cat# RHS4346
GIPZ Human ATF5 shRNA	Dharmacon	Cat# RHS4430-200253381

**Software and algorithms**		

FIJI software	National Institute of Health	https://fiji.sc/
Image Lab software	BioRad	https://www.bio-rad.com/en-jp/product/image-lab-software?ID=KRE6P5E8Z
Gene Set Enrichment Analysis (GSEA)	UC San Diego	https://www.gsea-msigdb.org/gsea/index.jsp
Harmony Software	Perkin Elmer	ver. 4.9
Excel software	Microsoft	https://www.microsoft.com/en-us/microsoft-365/excel
R software	The R Foundation	https://www.r-project.org/

### Experimental model and study participant details

#### Cell culture

The KP4 human pancreatic ductal cell carcinoma cells (Male, RCB1005, Riken Cell Bank), A549 lung cancer cells (Male, CCL-185, American Type Culture Collection), and SUIT2 human pancreatic cancer cells (Male, JCRB1094, JCRB cell bank) were cultured in Dulbecco’s Modified Eagle Medium (D6046, Sigma-Aldrich or 08456-65, Nacalai Tesque) supplemented with 10% fetal bovine serum (172012, Sigma-Aldrich) and 1% antibiotic/antimycotic solution (A5955, Sigma-Aldrich). The AsPC1 human pancreatic adenocarcinoma cells (Female, CRL-1682, American Type Culture Collection) were cultured in RPMI 1640 Medium (11875-119, Gibco) supplemented with 10% fetal bovine serum (172012, Sigma Nichirei Biosciences) and 1% antibiotic/antimycotic solution (A5955, Sigma-Aldrich). The HEK293 cells (embryo, RCB1637, Riken Cell Bank) were cultured in Dulbecco’s Modified Eagle Medium (D5796, Sigma-Aldrich) supplemented with 10% fetal bovine serum. Cells were cultured at 37°C in a humidified incubator with 5% CO_2_. All cells were tested for mycoplasma contamination using a MycoAlert Assay Control Set (LT07-518, Lonza Bioscience) and not authenticated.

We constructed the pIRES2-*EGR1*-ZsGreen1 vector to establish *EGR1*–overexpressed cells. For purposes of construction, we cloned the two fragments of CDS of *EGR1* sequence from cDNA of A549 cells via PCR with KOD Plus *Neo* (KOD-401, TOYOBO) with the primers (fragment-1 forward: 5′-ATGGCCGCGGCCAAGGCCGAG-3′, reverse: 5′-GCTCTCCAGGCCCTGGAAGGG-3’; fragment-2 forward: 5′-CGCACCCAGCAGCCTTCGCTAACC-3′, reverse: 5′-TTAGCAAATTTCAATTGTCCTGGG-3′). Next, the fragments were purified with NucleoSpin Gel and PCR Clean-up (U0609B, Takara) and used for PCR with KOD Plus *Neo* with the primers (fragment-1 forward: 5′-CGTCAGATCCGCTAGGCCACCATGGCCGCGGCCAAGGCC-3′, reverse: 5′-AGGCTGCTGGGTGCGGCTCTCCAGGCCCTGGAA-3’; fragment-2 forward: 5′-CGCACCCAGCAGCCTTCG-3′, reverse: 5′-GTCGACTGCAGAATTTTAGCAAATTTCAATTGTCCTG-3′). Then, the fragments were purified with NucleoSpin Gel and PCR Clean-up and inserted it into multicloning site of pIRES2-ZsGreen1 (632478, Clontech) digested with NheI-HF (R3131S, New England Biolabs) and EcoRI-HF (R3101S, New England Biolabs) by using In-Fusion HD Cloning Kit (Z9649N, Takara). The construction was confirmed by diagnostic digestion and sequencing. KP4 cells transfected with pIRES2-ZsGreen1 or pIRES2-*EGR1*-ZsGreen1 by TransIT-X2 Dynamic Delivery System (V6103, Takara) were selected with G-418 sulfate (4 mg/mL, V7983, Promega).

The non-silencing control shRNA-transgenic (shNC) and sh*ATF5*-transgenic (sh*ATF5*) KP4 cells were established by lentiviral transfection of GIPZ Non-silencing Lentiviral shRNA Control (RHS4346, Dharmacon) and GIPZ Human *ATF5* shRNA (RHS4430-200253381, Dharmacon), respectively. HEK293 cells were used for viral production. Virus-transfected cells were selected by culturing with puromycin (2 μg/mL, P9620, Sigma-Aldrich). The expression of fluorescent proteins in the transgenic cells was confirmed using the ZOE Fluorescent Cell Imaging System (BioRad).

The following inhibitors and antibodies were used for cell culture: Dimethyl Sulfoxide (DMSO, 1:1,000, 046–21981, Wako), JAK inhibitor I (5 μM, 420099, Sigma-Aldrich), MYCi361 (5 or 10 μM, T12132, TargetMol), Latrunculin A (0.1 μM, 125–04363, Wako), Blebbistatin (17 μM, BML-EI315-0005, Enzo Life Sciences), control rat IgG (2 μg/mL, I8015, Sigma-Aldrich or 147–09521, Wako), AIIB2 (2 μg/mL, Developmental Studies Hybridoma Bank at the University of Iowa), Y27632 (10 μM, Y0503, Sigma-Aldrich), PF-562271 (10 μM, PZ0387, Sigma-Aldrich), and Calyculin A (2 nM, 208851, Sigma-Aldrich).

#### Substrates

For collagen coating, plastic or glass dishes were treated with 0.3 mg/mL Cellmatrix Type I-C (Nitta Gelatin) or 0.5 mg/mL Atelocollagen Acidic Solution (IPC-50, KOKEN). To prepare collagen gel substrates, plastic or glass dishes were filled with neutralized collagen solution (1.6 mg/mL, Cellmatrix Type I-P, Nitta Gelatin) and incubated at 37°C for 30 min for gelation. Genipin-mixed collagen gels were prepared as previously reported.^40^ Briefly, equal volumes of genipin premix solution (x2 genipin concentration of final collagen gel, 100 mM HEPES, 16 g/L NaCl, 2.3 g/L Na_2_HPO_4_, 0.4 g/L KCl, 0.4 g/L KH_2_PO_4_, pH = 7.3–7.4) and Atelocollagen Acidic Solution were mixed well with gentle pipetting for 60 s on ice. The mixture was then quickly centrifuged to remove bubbles. The solution was poured on an iced glass dish and incubated at 37°C for 72 h for gelation. The gels were washed with phosphate-buffered saline (PBS) for 1 h thrice, DMEM for 24 h, and DMEM supplemented with 10% fetal bovine serum and 1% antibiotic/antimycotic solution for 24 h at 37°C. The polyacrylamide gels for cell culture were prepared as previously reported.^75^ The polyacrylamide gels were prepared using the following reagents (0.4 kPa: 0.05% N,N′-methylenebisacrylamide (BIS) and 5.0% acrylamide; 271 kPa: 0.6% BIS and 12% acrylamide).

#### Human samples

Tissue samples of human pancreatic ductal adenocarcinoma were obtained at the time of surgery from patients who had provided informed consent. This study was conducted in accordance with the Helsinki Declaration for Human Research and approved by the Ethics Committee of Nagoya University Graduate School of Medicine (approval number: 2017-0127-04). Clinical and demographic details of patients are shown in Table S1.

#### Subcutaneous pancreatic cancer mouse model

All animal protocols were reviewed and approved by the Animal Care and Use Committee of Nagoya University Graduate School of Medicine (approval numbers 30366 and 20409), and studies were conducted in compliance with institutional and national guidelines. In the transplantation model of mouse mT5 pancreatic cancer cells (generously provided by David Tuveson (Cold Spring Harbor Laboratory)), 1 × 10^6^ cells were transplanted subcutaneously into the backs of 8-week-old C57BL/6 female mice (RRID:IMSR_JAX:000664), followed by daily oral administration of saline or AM80 (3 mg/kg/day, 3507, Tocris), as described previously.^9^ Mice were euthanized on day 14 after tumor implantation, following which tumors were harvested, sliced, and immediately fixed in a formalin solution for subsequent histological analysis.

#### Tumor transplantation mouse model

5.0 × 10^6^ human KP4 pancreatic cancer cells (shNC or sh*ATF5*) were transplanted subcutaneously into the backs of 7-weeks-old Nude(BALB/c-nu) female mice (7 mice for shNC KP4 cells and 8 mice for sh*ATF5* KP4 cells). Tumor sizes were measured using calipers 30 days after transplantation, and tumor volumes were calculated using the formula X^2^ × Y × 0.5 (where X is the smaller diameter and Y is the larger diameter). Subsequently, the mice were sacrificed, and the tumors were excised and weighed to determine tumor weight.

### Method details

#### GEPIA2 database analysis

The GEPIA2 (Gene Expression Profiling Interactive Analysis, http://gepia2.cancer-pku.cn/#index) database was used to compare the expression levels of *ATF5, CTNNB1, RELA, TWIST1*, and *YAP1* in pancreatic adenocarcinoma, lung adenocarcinoma, breast invasive carcinoma (including subtypes), bladder urothelial carcinoma, and ovarian serous cystadenocarcinoma with corresponding normal tissues. We input the following values for differential analysis: Log2FC-cutoff (0.58) and *p* value (0.05).

#### Immunofluorescent staining

KP4, SUIT2 cells (2x10^4^ cells) or A549, AsPC1 cells (1x10^4^ cells) were seeded on 220 μL of collagen gels in 16 mm glass dishes or collagen-coated 16 mm glass dishes and cultured for 48 h. KP4 or A549 cells (2x10^5^ cells) were seeded on 24 × 24 mm^2^ polyacrylamide gels and cultured for 48 h. For the treatment of DMSO, JAK inhibitor I, Latrunculin A, Blebbistatin, Y27632, PF-562271, control IgG, or AIIB2, KP4 cells (1x10^4^ cells) were seeded on 220 μL of collagen gels in 16 mm glass dishes or collagen-coated 16 mm glass dishes and cultured for 24 h. The cells were then treated with the reagents and cultured for 48 h. For the treatment of MYCi361 or corresponding control DMSO, KP4 cells (2x10^4^ cells) were seeded on collagen-coated 16 mm glass dishes and cultured for 24 h. Then, the cells were treated with the reagents and cultured for 24 h. For the treatment of Calyculin A or corresponding control DMSO, KP4 cells (2x10^4^ cells) were seeded on 220 μL of collagen gels in 16 mm glass dishes and cultured for 24 h. Then, the cells were treated with the reagents and cultured for 24 h. The cells were fixed with 4% paraformaldehyde in PBS for 10 min at room temperature and washed thrice with PBS. Next, the cells were permeabilized with 0.5% Triton X-100 in PBS for 10 min at room temperature and washed thrice with PBS. The cells were blocked with 5% (for ATF5) or 0.5% (for PP-MRLC) skimmed milk in PBS for 30 min (for ATF5) or 1 h (for PP-MRLC) at room temperature. The primary antibody solution (1:200 Anti-ATF5, Rabbit-Mono(SD2099), NBP2-67767, Novus Biologicals) in PBS or (1:200 Anti-PP-MRLC, Phospho-Myosin Light Chain 2 (Thr18/Ser19) Antibody, 3674S, Cell Signaling Technology) in 0.5% skimmed milk in PBS was added and incubated at 4°C overnight. After three washes with PBS (for ATF5) or 0.5% skimmed milk in PBS (for PP-MRLC), the secondary antibody solution (1:200 Goat anti-Rabbit IgG(H + L), Superclonal Recombinant Secondary Antibody, Alexa Fluor 488; A27034, Invitrogen; 1:4,000 Hoechst 33342, H1399, Invitrogen; with or without 1:500 Alexa Fluor 546 Phalloidin, A22283, Invitrogen) in PBS or (1:500 Goat anti-Rabbit IgG(H + L), Superclonal Recombinant Secondary Antibody, Alexa Fluor 488; 1:4,000 Hoechst 33342; 1:500 Alexa Fluor 546 Phalloidin) in 0.5% skimmed milk in PBS was added and incubated at room temperature for 1 h. After washing thrice with PBS (for ATF5) or 0.5% skimmed milk in PBS (for PP-MRLC), fluorescent images were captured using a C2 confocal imaging system (Nikon Instech) with a 60× objective. We avoided capturing images of the cells on the gel at the center of the dish because the gel at the center was considerably thin. To quantify the localization of ATF5, fluorescence intensity was calculated as the nuclear/cytoplasm ratio using FIJI software (National Institute of Health).

#### Western blotting

KP4 or A549 cells (5x10^4^ cells) were seeded on 500 μL of collagen gel in 35 mm plastic dishes or collagen-coated 35 mm plastic dishes and cultured for 48 h. For treatment of JAK inhibitor I or corresponding control DMSO, KP4 cells (5x10^4^ cells) were seeded on collagen-coated 35 mm plastic dishes and cultured for 24 h. Then, the cells were treated with the reagents and cultured for 24 h. For treatment of Latrunculin A, Blebbistatin, control IgG, AIIB2 or corresponding control DMSO, KP4 cells (5x10^4^ cells) were seeded on collagen-coated 35 mm plastic dishes and cultured for 24 h. Then, the cells were treated with the reagents and cultured for 48 h.

Proteins were extracted from the cells on collagen-coated plastic dishes or collagen gels according to a previous report of ours^78^ with some modifications. The cells on collagen-coated plastic dishes, were treated with ice cold 10% trichloroacetic acid (TCA) in PBS and incubated for 3 min on ice. After washing thrice with PBS, proteins were extracted from the cells using sodium dodecyl sulfate (SDS) sample buffer (0.125 M Tris HCl (pH = 6.8), 2.3% SDS, 10% glycerol, and 5% dithiothreitol, 0.01% bromophenol blue). The samples were then sonicated and boiled for 5 min. The cells on collagen gels were treated with ice cold 10% TCA in PBS and incubated for 15 min on ice. After washing thrice with PBS, 0.1% collagenase (034–22363, Wako) in PBS was added and incubated for approximately 5 min at 37°C. After collagen digestion, collagenase was removed via centrifugation at 10,000 rpm for 5 min. Proteins were extracted from the cells using SDS sample buffer. Then, the samples were sonicated and boiled for 5 min.

We used 10% (ATF5, EGR1, MYC, and corresponding control β-actin) or 8% (pJAK and corresponding control β-actin) polyacrylamide gels for SDS-PAGE (20 mA per gel). After SDS-PAGE, blotting to polyvinylidene difluoride membranes was performed (92 mA per gel). After the blotting, the membranes were incubated in 5% skim milk (ATF5, pJAK, MYC, or β-actin) or 5% bovine serum albumin (EGR1) in Tris-buffered saline plus Tween 20 (TBS-T) for 30 min (ATF5, EGR1, pJAK, or β-actin) or 1 h (MYC and corresponding control β-actin) at room temperature. The membranes were incubated with primary antibodies (150,000 Anti-ATF5, Rabbit-Mono(SD2099); 1:3,000 EGR1(15F7) Rabbit mAb, 4153S, Cell Signaling Technology; 1:10,000 Anti-*c*-Myc-ChIP Grade, ab32072, abcam; or 1:10,0000 β-actin Anti-beta actin antibody (AC-15), ab6276, abcam) in TBS-T or primary antibody (Phospho-Jak1 (Tyr1034/1035) (pJAK) Antibody, 3331S, Cell Signaling Technology; or 1:1,000 Myc Antibody (9E10), sc-40, Santa Cruz) in Can Get Signal Solution 1 (NKB-101, TOYOBO) at 4°C overnight. After washing thrice with TBS-T, the membranes were incubated with secondary antibodies (1:100,000 Anti-mouse IgG, HRP-linked Antibody, 7076S, Cell Signaling Technology; or 1:10,000 Anti-rabbit IgG, HRP-linked Antibody, 7074S, Cell Signaling Technology) in TBS-T (ATF5, EGR1, MYC, or β-actin) or Can Get Signal Solution 2 (NKB-101, TOYOBO) (pJAK) for 1 h at room temperature. For detection of MYC with Myc Antibody (9E10), the membranes were incubated with secondary antibodies (1:10,000 Anti-mouse IgG, HRP-linked Antibody) in Can Get Signal Solution 2 for 1 h at room temperature. After washing thrice with TBS-T, Signals were detected with Immobilon Western Chemiluminescent HRP substrate (WBKLS0500, Millipore) and ChemiDoc Touch Imaging System (BioRad). The relative intensity of proteins was calculated by normalizing the intensity of target proteins with the intensity of β-actin analyzed via Image Lab software (BioRad).

#### siRNA transfection

KP4 (2x10^5^ cells) or A549 cells (1x10^5^ cells) were seeded on 35 mm plastic dishes and cultured for 24 h. Then, cells were transfected with 8 pmol siRNA or a negative control non-targeting RNA duplex via the Lipofectamine RNAiMAX Transfection Reagent (13778150, Invitrogen). After 24 h, the cells were harvested, seeded on collagen gels in plastic dishes or collagen-coated plastic or glass dishes, and used for subsequent experiments. The siRNA duplex was synthesized using an *in vitro* Transcription T7 kit (6140, TAKARA) and Klenow Fragment (3'→5' exo-) (M0212L, New England Biolabs). The target sequences (5′ to 3′) were as follows: non-targeting (antisense: AAACTACATGTCACATCACGG, sense: AACCGTGATGTGACATGTAGT), si*KPNB1*-1 (ATCCATGAATTGTTAACTGAA), si*KPNB1*-2 (ATCTAAAGATCCAGATATCAA), si*KPNB1*-3 (TACAAATCAAGAACTCTTTGA), si*ATF5*-1 (TCGTCCAAATCATGAAATGTT), si*ATF5*-2 (AGCAAAAATAAAACGAAACAT), si*JAK1* (AGCAATGAAGTTGCCATTTAA), si*JAK2* (AGCCATCATACGAGATCTTAA), si*TYK2* (TGCTATATTTCCGCATAAGGT), si*MYC* (AACGTTTATAGCAGTTACACA), si*YAP1* (AACGTTTATAGCAGTTACACA), si*EGR1* (TACTCTTGATGTGAAGATAATTT), si*RAC1*-1 (AACTATGAACCTTCTTAACATCA), si*RAC1*-2 (AGCAATATGCCTCCTTGTATTAT).

#### RNA extraction

KP4 or A549 cells (5x10^4^ cells) were seeded on 500 μL of collagen gels in 35 mm plastic dishes or collagen-coated 35 mm plastic dishes and cultured for 48 or 72 h. For the treatment of MYCi361 or corresponding control DMSO, KP4 cells (5x10^4^ cells) were seeded on collagen-coated 35 mm plastic dishes and cultured for 24 h. Then, the cells were treated with the reagents and cultured for a further 24 h. For the treatment of JAK inhibitor I, Latrunculin A, Blebbistatin, control IgG, AIIB2 or corresponding control DMSO, KP4 cells (5x10^4^ cells) were seeded on collagen-coated 35 mm plastic dishes and cultured for 24 h. Then, the cells were treated with the reagents and cultured for 48 h. To compare the cells on collagen gels with the cells on collagen-coated plastic dishes, we extracted RNA with TriPure Isolation Reagent (11667165001, Roche) followed by purification with Fast Gene RNA Premium kit (FG-81050, NIPPON kkkGenetics). On other cases, RNA was extracted by FastGene RNA Basic Kit (FG-80250, NIPPON Genetics).

#### qPCR

First, cDNA synthesis was performed using ReverTra Ace qPCR RT Master Mix (FSQ-201, TOYOBO). Quantitative PCR (qPCR) was performed by KAPA SYBR Fast qPCR kit (KK4602, NIPPON Genetics) and StepOnePlus (Thermo Scientific). The relative expression of mRNA was calculated by normalizing the expression of target mRNA with the expression of β-actin (*ACTB*). The following primers were used: (5′ to 3′): β-actin (*ACTB*) (Forward: TGGGACGACATGGAGAAAATCTG, Reverse: AGGTCTCAAACATGATCTGGGTC), KPNB1 (Forward: TGGCTTCAAATGTGTGCTGGG, Reverse: TGGTGTCCATCAGGTCTGTCTG), Ki67 (*MKI67*) (Forward: ATCGTCCCAGTGGAAGAGTTG, Reverse: TCGACCCCGCTCCTTTTGATAG), *SLCO2A1* (Forward: TGCCAACTTCCTCATTGGTGC, Reverse: ATGCGGGGAATGGCTTGTAG), *PTPN13* (Forward: AGCTTGGGGATAAGTGTCACGG, Reverse: AGCTAGGACGCGATCACCTTTG), *IFIT1* (Forward: AGGCCTGCTTTGAAAAGGTGC, Reverse: TTAAGCGGACAGCCTGCCTTAG), *EGR1* (Forward: CCTAAGCTGGAGGAGATGATGC, Reverse: TGTCAGGAAAAGACTCTGCGG), *NR4A2* (Forward: AAACAAAGGCACATTGGCGGC, Reverse: CATTCAACTCTGCCGAAGTGCAG), *CD55* (Forward: ACTTCCAAGGTCCCACCAACAG, Reverse: GGTGTACTCCGTGTTGCTTGAG), *ID2* (Forward: ATCCTGTCCTTGCAGGCTTC, Reverse: ACCGCTTATTCAGCCACACAG), *PRKCQ*-V1,2,4,5 (Forward: AACTTTGACTGCGGGTCCTG, Reverse: ATCTGCCCGTTCTCTGATTCG), *PRKCQ*-V3,6,7 (Forward: TGTCGCCATTTCTTCGGATTGG, Reverse: ATACATCTGCCCGTTCTGATTCG), *CFP54*-V1 (Forward: CAGAATACAGCCGAGCCAAAGC, Reverse: GCTGGCTTGGAGAACATCTTGTG), *CFAP54*-V2 (Forward: ATTTTGCCTGGGGACAGAGGAG, Reverse: TCCAAGTGAATGCAACGCCTG), *PIFO*-V1 (Forward: TTTTTCCTCACTTCCATCCCCCG, Reverse: CTGCATGAGGTGTCATTTCCTTCTG), *PIFO*-V2 (Forward: TCACGGGACTAGCCTTCGTTTC, Reverse: TAGTTATCCGCAGCGTCTGCTC), *PIFO*-V1,2 (Forward: ACCCTAAAAGCTGAGCTGTCCAC, Reverse: AGACAAGAGGGCCACAGTAACG), *ATF5* (Forward: CCTATGAGGTCCTTGGGGGA, Reverse: GAGGCCATAGCTTCCAGGTC), *JAK1* (Forward: GTCACCTGCTTTGAGAAGTCTGAGC, Reverse: ACCAGCAGGTTGGAGATTTCTCGG), *JAK2* (Forward: TGACTTTTGCTGTCGAGCGAG, Reverse: TTTTGGCTTTGGGGGACAGC), *TYK2* (Forward: TGGTCAAGATCGGGGACTTTGG, Reverse: ACTCTGGGGCATACCAGAACAC), *MYC* (Forward: GCGACTCTGAGGAGGAACAAGAAG, Reverse: GTTGTGCTGATGTGTGGAGACG), *YAP1* (Forward: ATCCTTTCCTTAACAGTGGCACC, Reverse: GGCAGGGTGCTTTGGTTGATAG), *RAC1* (Forward: TGGCTAAGGAGATTGGTGCTG, Reverse: AGCGTACAAAGGTTCCAAGGG), *CCN2* (Forward: CCTGGAAGAGAACATTAAGAAGGGC, Reverse: AATGGCAGGCACAGGTCTTG), *GADD45B* (Forward: CATTGTCTCCTGGTCACGAACC, Reverse: AATGGCAGGCACAGGTCTTG), *AXL* (Forward: ACCAGCAAGAGCGATGTGTG, Reverse: GCGACATCAAGGCATACAGTCC), *TGM2* (Forward: TGGTGAGTGGCATGGTCAAC, Reverse: ATGGGCCGAGTTGTAGTTGG), *DAB2* (Forward: TGGGACTTTGAGTGCCTTTGC, Reverse: AGCTGGCTTTGGTGGTTCATTG), *LHFPL6* (Forward: TAGGGACAGAGGCAAAGAAGGG, Reverse: TAAAGATGATGGTCCATGGCTGG), *DDAH1* (Forward: GCGGAGATGTTTTATTCACAGGCAG, Reverse: ATTGCGATCAGGTTAGGCCCAG), *THBS1* (Forward: AGTTTGGAGGCAAGGACTGC, Reverse: ACAGGCATCCATCAATTGGACAG).

#### Microarray and GSEA

A DNA microarray (Clariom S Array, Human; Applied Biosystems) assay was performed using RNA extracted from the cells prepared according to the procedure described in the RNA extraction section. The data were analyzed by Gene Set Enrichment Analysis (GSEA, https://www.gsea-msigdb.org/gsea/index.jsp).

#### Cell growth assay

KP4 or A549 cells (5x10^4^ cells) were seeded on 500 μL of collagen gels in 35 mm plastic dishes or collagen-coated 35 mm plastic dishes and cultured for 48 h. Phase-contrast images were captured using a TE300 microscope (Nikon Instech) equipped with a 10× objective. For the cells on collagen-coated dishes, the media in the dish was transferred to a tube. The cells were washed twice with PBS. The PBS after wash was also transferred to the same tube. Then, the cells were treated with Trypsin-EDTA (0.25%), phenol red (25200-072, Gibco) for 5 min at 37°C. The cells were suspended and transferred to the same tube. The mixture in the tube was centrifuged at 1,000 rpm for 2 min. After removal of the supernatant, the cell pellet was suspended with 250 μL of culture media. For the cells on collagen gels, the media in the dish was transferred to a tube. The cells were treated with 0.1% collagenase in PBS and incubated at 37°C until the gel was completely dissolved and the cells were suspended. Then, the suspension was transferred to the same tube and centrifuged at 1,000 rpm for 2 min. At the same time, Trypsin-EDTA (0.25%), phenol red was added to the dish and incubated for 5 min at 37°C. After removal of the supernatant from the tube, the Trypsin-EDTA solution was added to the tube, suspended, and incubated within 5 min. Next, the suspension was centrifuged at 1,000 rpm for 2 min. After removal of the supernatant, the cell pellet was suspended with 250 μL of culture media.

Ten microliters (10 μL) of the suspension was mixed with the same volume of Trypan Blue Stain (0.4%) for use with a Countes Automated Cell Counter (T10282, Invitrogen). The total cell number and dead cell (Trypan Blue-positive cell) number were counted using a hemocytometer. The relative cell number was calculated as a ratio of the total cell number. The proportion of survival cells was calculated by (total cell number – dead cell number)/total cell number.

#### Drug screening

KP4 cells (3.2x10^3^ cells) were seeded on collagen-coated 96 well glass plates and cultured for 24 h. The cells were treated with the reagents (1:1,000, custom 500 drug library, TargetMol) or 1:1,000 DMSO and cultured for 24 h. Then, fluorescent staining was performed, and images were captured and analyzed via Operetta CLS (5250040, PerkinElmer) and Harmony Software (ver. 4.9, PerkinElmer), respectively. The candidate drugs regulating ATF5 localization to nuclei were obtained via the following procedures: first, the drugs which decreased the cell number (<0.5, relative to DMSO = 1) were excluded; second, the drugs which decreased (<0.85) or increased (>1.15) ATF5 intensity (nuclei/cytoplasm, relative to DMSO = 1) were extracted and selected as candidate drugs (Figure S4A).

#### Immunoprecipitation

KP4 cells (2x10^6^ cells) were seeded on collagen-coated 100 mm plastic dishes and cultured for 48 h. KP4 cells (2x10^5^ cells) were seeded on 50 × 70 mm^2^ polyacrylamide gels and cultured for 48 h. We used Capturem IP&Co-IP kit (635721, Clontech) to prepare immune complexes. The cell lysates were incubated with Anti-ATF5, Rabbit-Mono(SD2099) or Normal Rabbit IgG, Whole Molecule, Purified (148–09551, Wako) at 4°C overnight. The immune complexes were extracted using SDS sample buffer and equilibrated with 1 M Tris-HCl (pH = 8.0) buffer. Then, the samples were sonicated and boiled for 5 min, followed by western blotting.

#### Immunohistochemistry

Formalin-fixed paraffin-embedded tissue sections were deparaffinized and rehydrated, followed by antigen retrieval by boiling the samples in Target-Retrieval Solution (Dako) at pH 9 for 30 min. After incubation with blocking solution for 10 min at room temperature, the samples were incubated with primary antibodies overnight at 4°C. After washing, the slides were incubated with horseradish peroxidase (HRP)-polymer secondary antibody ImmPRESS reagent HRP goat anti-rabbit IgG (MP-7451, Vector Laboratories, Newark, CA, USA) for 30 min at room temperature. The sections were washed and incubated with Liquid DAB+ Substrate Chromogen System (K3468, Dako) for 5 min at room temperature, followed by counterstaining with hematoxylin. Finally, the dehydrated and cleared tissues were mounted using Entellan mounting media (100869, Merck, Darmstadt, Germany). The antibodies used in the study included rabbit anti-ATF5 antibody (NBP2-67767, Novus Biologicals, dilution 1:400) and rabbit anti-EGR1 antibody (4153, Cell signaling technology, dilution 1:400).

### Quantification and statistical analysisis

To compare a value showing variance to another value with no variance, we determined significance via the 95% confidence interval. To compare between two values with variance, we verified the normality of the datasets using the Kolmogorov-Smirnov test, where *p* < 0.05 indicated that the data were not normally distributed Then, we determined whether the variance of two datasets was significantly different by using the F-test, in which *p* < 0.05 indicated that the variance was significantly different. We analyzed the significance of datasets without statistically different variances, using the two-sided Student’s t test. By contrast, for datasets with statistically different variances, we used two-sided Welch’s t-test to analyze statistical significance. For datasets including data that did not meet the normality assumption, we used the Wilcoxon rank-sum test to analyze statistical significance. Statistical significance for the Student’s t test, Welch’s t-test, and Wilcoxon rank-sum test, was set at *p* < 0.05. For multiple comparisons, we analyzed significance using the Bonferroni correction. For human tissue samples, statistical significance was determined via the paired t-test. For mice tumor samples, statistical significance was determined via Wilcoxon rank-sum test. All analyses were performed using Excel software (for confidence interval, F-test, Student’s t test, Welch’s t-test, and paired t-test) and R software (for Kolmogorov-Smirnov test and Wilcoxon rank-sum test). No statistical methods were performed to pre-determine the appropriateness of sample size.
