# Supplementary material for: Stiff extracellular matrix activates the transcription factor ATF5 to promote the proliferation of cancer cells
Source: iScience. 2025 Feb 17;28(3):112057. doi: 10.1016/j.isci.2025.112057 (PMC11928855; doi:10.1016/j.isci.2025.112057)
Supplement: Document S1. Figures S1–S7 and Table S1 [file mmc1.pdf]

iScience, Volume *28*

Supplemental information

Stiff extracellular matrix activates the transcription factor ATF5 to promote the proliferation of cancer cells

Seiichiro Ishihara, Atsushi Enomoto, Akihiro Sakai, Tadashi Iida, Shoichiro Tange, Noriyuki Kioka, Akihiro Nukuda, Ayaka Ichikawa Nagasato, Motoaki Yasuda, Takashi Tokino, and Hisashi Haga

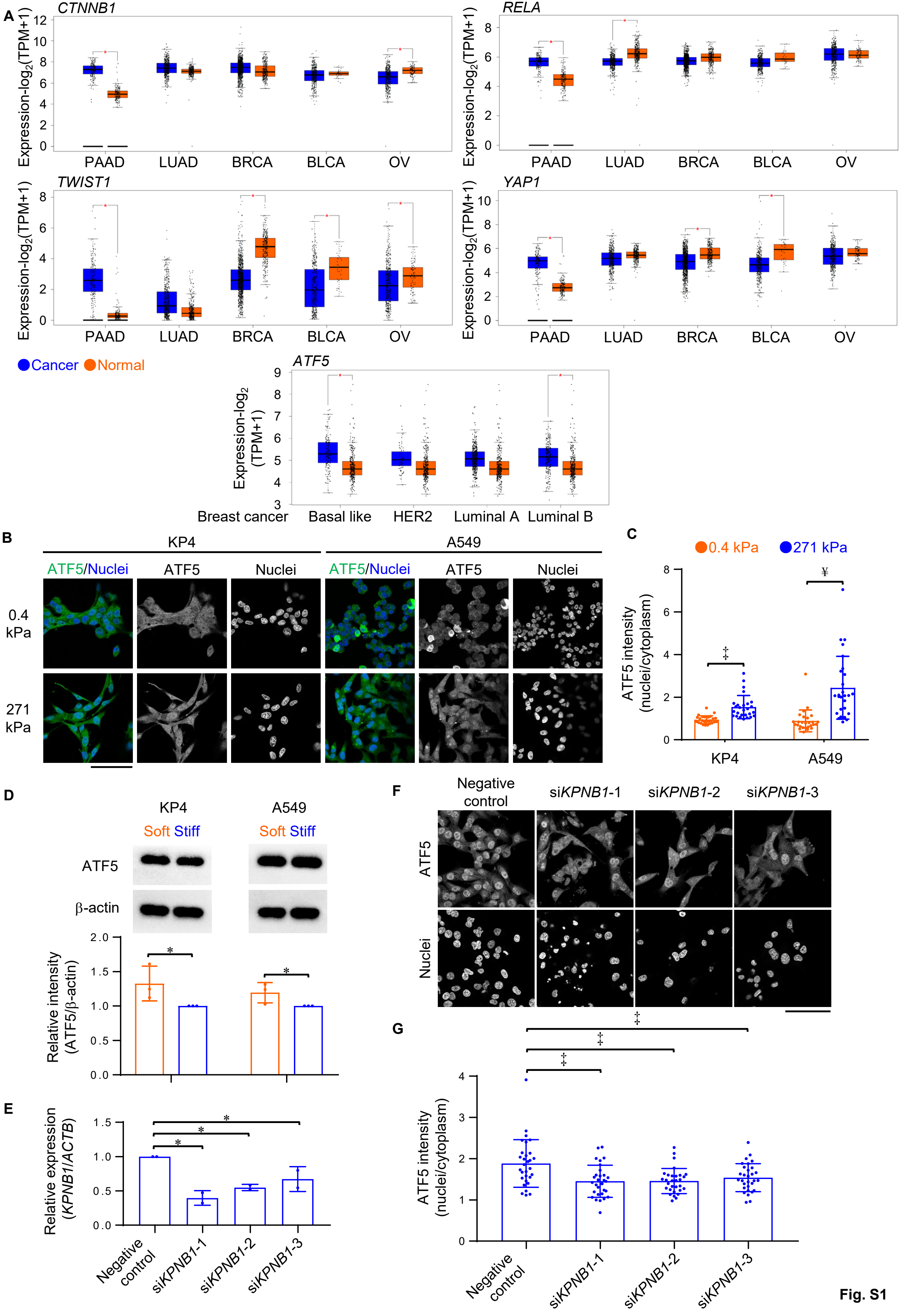
A
CTNNB1
RELA
TWIST1
YAP1
Expression-log2(TPM+1)
PAAD
LUAD
BRCA
BLCA
OV
Cancer
Normal
ATF5
Expression-log2 (TPM+1)
Breast cancer
Basal like
HER2
Luminal A
Luminal B
B
KP4
A549
ATF5/Nuclei
ATF5
Nuclei
0.4 kPa
271 kPa
C
0.4 kPa
271 kPa
ATF5 intensity (nuclei/cytoplasm)
KP4
A549
D
KP4
A549
Soft Stiff
Soft Stiff
ATF5
β-actin
Relative intensity (ATF5/β-actin)
E
Relative expression (KPNB1/ACTB)
Negative control
siKPNB1-1
siKPNB1-2
siKPNB1-3
F
Negative control
siKPNB1-1
siKPNB1-2
siKPNB1-3
ATF5
Nuclei
G
ATF5 intensity (nuclei/cytoplasm)
Negative control
siKPNB1-1
siKPNB1-2
siKPNB1-3

Fig. S1

Figure S1. ATF5 is highly expressed in cancer tissues and localizes in the nuclei by stiff matrices.

(A) *CTNNB1, RELA, TWIST1*, and *YAP1* mRNA levels in pancreatic adenocarcinoma (PAAD), lung adenocarcinoma (LUAD), breast invasive carcinoma (BRCA), bladder urothelial carcinoma (BLCA), and ovarian serous cystadenocarcinoma (OV) with corresponding normal tissues. *ATF5* mRNA levels in breast invasive carcinoma subtypes (Basal-like (triple negative), HER2+ non-luminal, Luminal A, and Luminal B), with corresponding normal tissues.

(B) Immunofluorescent staining of ATF5 and nuclei in KP4 and A549 cells on 0.4 kPa or 271 kPa polyacrylamide gel substrates.

(C) Relative intensity of ATF5 in the nuclei to that in the cytoplasm, quantified from (B); n = 27 cells in 3 experiments.

(D) Western blot of ATF5 and β-actin in KP4 and A549 cells on collagen gel (soft) or collagen-coated plastic (stiff) substrates. Relative intensity of ATF5 to β-actin is shown; n = 3 experiments.

(E) qPCR of *KPNB1* (an critical factor for active transport of proteins to nuclei) in KP4 cells transfected with negative control RNA, si*KPNB1*-1, si*KPNB1*-2, or si*KPNB1*-3 on collagen-coated plastic dishes. β-actin (*ACTB*) was used as an internal control; n = 2 experiments.

(F) Immunofluorescent staining of ATF5 and nuclei in KP4 cells transfected with negative control RNA, si*KPNB1*-1, si*KPNB1*-2, or si*KPNB1*-3 on collagen-coated glass dishes.

(G) Relative intensity of ATF5 in the nuclei to that in the cytoplasm, quantified from (F); n = 30 cells in 2 experiments.

Scale bar = 100 μm; mean with S.D. and each data point is shown; *, statistical significance determined with 95% confidence interval; ‡, statistical significance ($P < 0.05$) determined with Welch's t-test; ¥, statistical significance ($P < 0.05$) determined with Wilcoxon rank-sum test. For multiple comparisons, we analyzed significance using the Bonferroni correction.

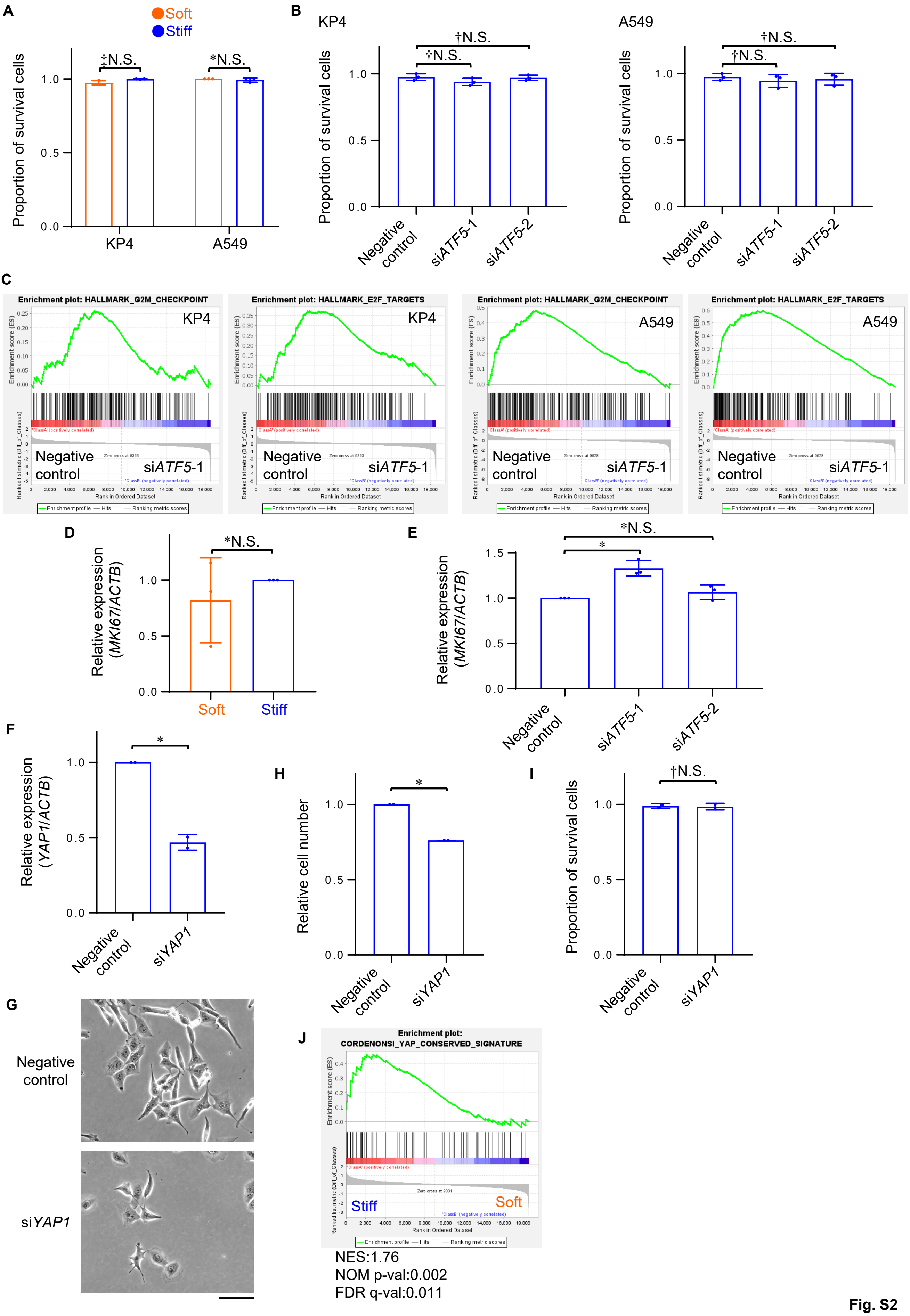
A
Soft
Stiff
Proportion of survival cells
‡N.S.
*N.S.
KP4
A549
B
KP4
A549
†N.S.
Negative control
siATF5-1
siATF5-2
C
Enrichment plot: HALLMARK_G2M_CHECKPOINT
Enrichment plot: HALLMARK_E2F_TARGETS
Negative control
siATF5-1
D
Relative expression (MKI67/ACTB)
E
F
Relative expression (YAP1/ACTB)
siYAP1
G
H
Relative cell number
I
J
Enrichment plot: CORDENONSI_YAP_CONSERVED_SIGNATURE
Stiff
Soft
NES:1.76
NOM p-val:0.002
FDR q-val:0.011

Fig. S2

Figure S2 Stiff matrix triggers proliferation of cancer cells via ATF5.

(A) Proportion of survival cells in KP4 or A549 cells on collagen gel (soft) or collagen-coated plastic (stiff) substrates; n = 3 experiments.

(B) Proportion of surviving cells in KP4 or A549 cells transfected with negative control RNA, si*ATF5*-1, or si*ATF5*-2 on collagen-coated plastic dishes; n = 3 experiments.

(C) GSEA of G2M checkpoint or E2F targets highlighted in Figure 2F (GSEA of upregulated genes for hallmark gene sets in KP4 or A549 cells transfected with negative control RNA or si*ATF5*-1 on collagen-coated plastic dishes).

(D) qPCR of Ki67 (*MKI67*) in KP4 cells on collagen gel (soft) or collagen-coated plastic (stiff) substrates. β-actin (*ACTB*) was used as an internal control; n = 3 experiments.

(E) qPCR of Ki67 (*MKI67*) in KP4 cells transfected with negative control RNA, si*ATF5*-1, or si*ATF5*-2 on collagen-coated plastic dishes. β-actin (*ACTB*) was used as an internal control; n = 4 experiments.

(F) qPCR of *YAP1* in KP4 cells transfected with negative control RNA or si*YAP1* on collagen-coated plastic dishes. β-actin (*ACTB*) was used as an internal control; n = 2 experiments.

(G) Phase-contrast images of KP4 cells transfected with negative control RNA or si*YAP1* on collagen-coated plastic dishes.

(H) Relative cell number, analyzed from (G); n = 2 experiments.

(I) Proportion of surviving cells, analyzed from (G); n = 2 experiments.

(J) GSEA of YAP conserved signature gene set in KP4 cells on collagen-coated plastic (stiff) substrates compared with the cells on collagen gel (soft) substrates or KP4 cells transfected with negative control RNA or siATF5-1 on collagen-coated plastic dishes. NES, normalized enrichment score; NOM p-val, nominal p value;. FDR q-val, false discovery rate q value.

Scale bar = 100 μm; mean with S.D. and each data point is shown; *, statistical significance determined with 95% confidence interval; †, statistical significance ($P < 0.05$) determined via Student's t-test; ‡, statistical significance ($P < 0.05$) determined via Welch's t-test; N.S., no significance with marked test. For multiple comparisons, we analyzed significance using the Bonferroni correction.

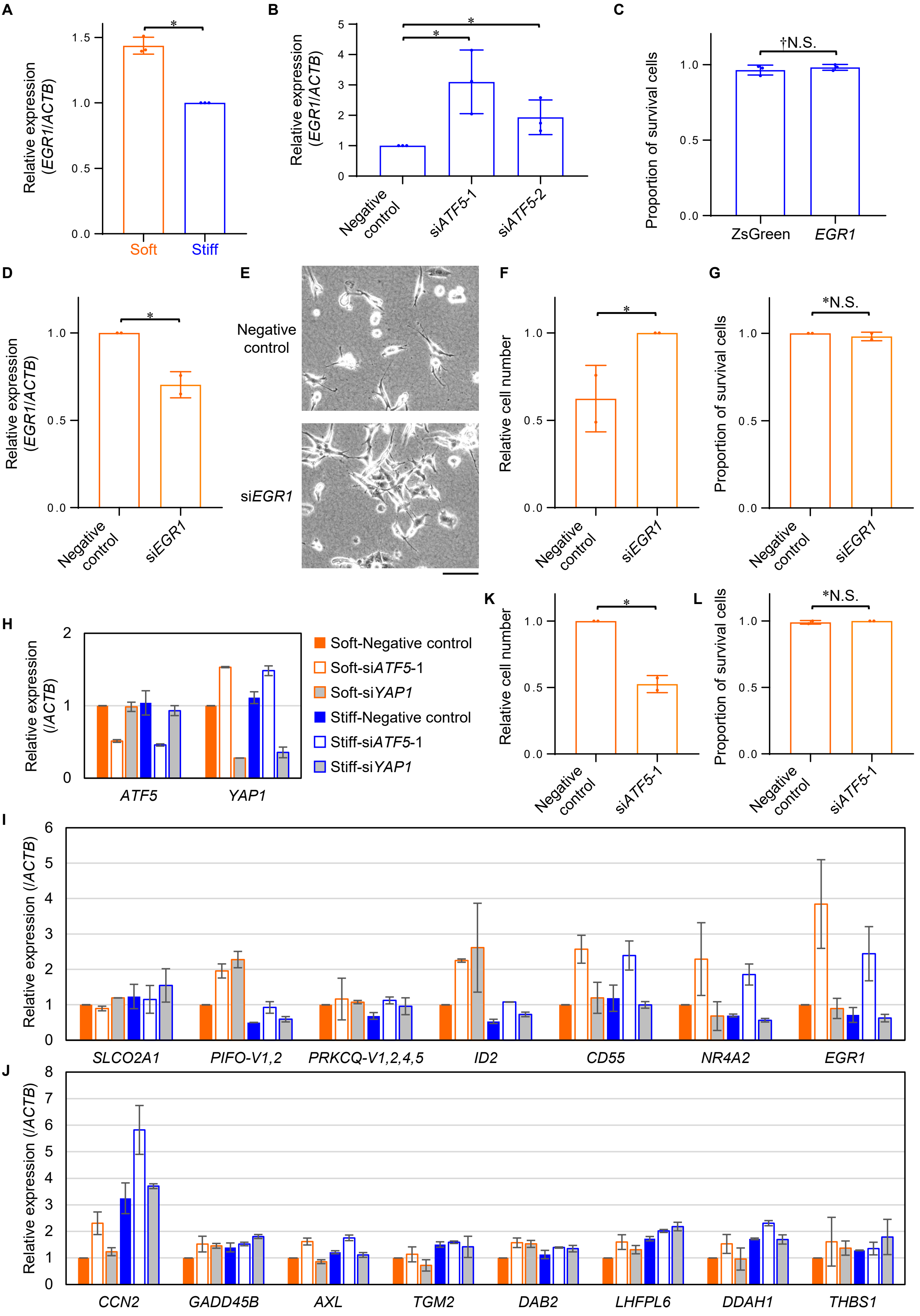
A
Relative expression (EGR1/ACTB)
Soft
Stiff
*
B
Relative expression (EGR1/ACTB)
Negative control
siATF5-1
siATF5-2
*
*
C
Proportion of survival cells
ZsGreen
EGR1
†N.S.
D
Relative expression (EGR1/ACTB)
Negative control
siEGR1
*
E
Negative control
siEGR1
F
Relative cell number
Negative control
siEGR1
*
G
Proportion of survival cells
Negative control
siEGR1
*N.S.
H
Relative expression (/ACTB)
ATF5
YAP1
Soft-Negative control
Soft-siATF5-1
Soft-siYAP1
Stiff-Negative control
Stiff-siATF5-1
Stiff-siYAP1
K
Relative cell number
Negative control
siATF5-1
*
L
Proportion of survival cells
Negative control
siATF5-1
*N.S.
I
Relative expression (/ACTB)
SLCO2A1
PIFO-V1,2
PRKCQ-V1,2,4,5
ID2
CD55
NR4A2
EGR1
J
Relative expression (/ACTB)
CCN2
GADD45B
AXL
TGM2
DAB2
LHFPL6
DDAH1
THBS1

Fig. S3

Figure S3. ATF5 activated by stiff matrix suppresses EGR1 expression.

(A) qPCR of *EGR1* in A549 cells on collagen gel (soft) or collagen-coated plastic (stiff) substrates. β-actin (*ACTB*) was used as an internal control; n = 3 experiments.

(B) qPCR of *EGR1* in A549 cells transfected with negative control RNA, si*ATF5*-1, or si*ATF5*-2 on collagen-coated plastic dishes. β-actin (*ACTB*) was used as an internal control; n = 3 experiments.

(C) Proportion of survival cells in KP4 cells transfected with ZsGreen or EGR1 vector for *EGR1* overexpression on collagen-coated plastic dishes; n = 3 experiments on collagen-coated plastic dishes.

(D) qPCR of *EGR1* in KP4 cells transfected with negative control RNA or si*EGR1* on collagen gel substrates. β-actin (*ACTB*) was used as an internal control; n = 2 experiments.

(E) Phase-contrast images of KP4 cells transfected with negative control RNA or si*EGR1* on collagen gel substrates.

(F) Relative cell number, analyzed from (E); n = 2 experiments.

(G) Proportion of surviving cells, analyzed from (E); n = 2 experiments.

(H) qPCR of *ATF5* and *YAP1* in KP4 cells transfected with negative control RNA, si*ATF5*-1, or si*YAP1* on collagen gel (soft) or collagen-coated plastic (stiff) substrates. β-actin (*ACTB*) was used as an internal control; n = 2 experiments.

(I) qPCR of genes regulated by stiffness and ATF5 in KP4 cells transfected with negative control RNA, si*ATF5*-1, or si*YAP1* on collagen gel (soft) or collagen-coated plastic (stiff) substrates. β-actin (*ACTB*) was used as an internal control; n = 2 experiments.

(J) qPCR of genes regulated by stiffness and YAP1 in KP4 cells transfected with negative control RNA, si*ATF5*-1, or si*YAP1* on collagen gel (soft) or collagen-coated plastic (stiff) substrates. β-actin (*ACTB*) was used as an internal control; n = 2 experiments.

(K) Relative cell number of KP4 cells transfected with negative control RNA or si*ATF5*-1 on collagen gel substrates; n = 2 experiments.

(L) Proportion of surviving cells in KP4 cells transfected with negative control RNA or si*ATF5*-1 on collagen gel substrates; n = 2 experiments.

Scale bar = 100 μm; mean with S.D. and each data point (A, B, C, D, E, F, G, K, L) or mean with S.D. (H, I, J) is shown; *, statistical significance determined with 95% confidence interval; †, statistical significance ($P < 0.05$) determined via Student's t-test; N.S., no significance with marked test. For multiple comparisons, we analyzed significance using the Bonferroni correction.

A

CAS	Drug name	Cell number (DMSO=1)	ATF5 intensity (nuclei/cytosol, DMSO=1)
288250-47-5	Y320	0.578	0.707
1675203-84-5	PD1-PDL1 inhibitor 2	0.521	0.727
1550371-22-6	BMS-P5 free base	0.771	0.776
96829-58-2	Orlistat	0.907	0.778
53716-50-0	Oxfendazole	0.503	0.779
6055-19-2	Cyclophosphamide monohydrate	0.979	0.784
1314118-94-9	MK2-IN-1 hydrochloride	0.617	0.797
81131-70-6	Pravastatin sodium	1.03	0.801
182004-65-5	KB-R7943	0.624	0.811
292053-42-0	CCI-006	0.757	0.827
65-49-6	Aminopar	0.994	0.835
73573-88-3	Mevastatin	0.639	0.839
1575596-29-0	PRN1008	0.725	1.158
501-36-0	Resveratrol	0.548	1.19
1421919-75-6	KPT276	0.646	1.207
2196204-23-4	AZ1495	0.548	1.244
1638211-04-7	ML303	0.985	1.268
1360614-48-7	(E)-Necrosulfonamide	0.66	1.43

B

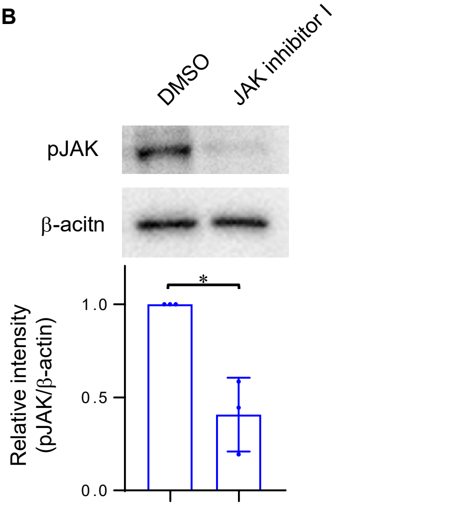

C

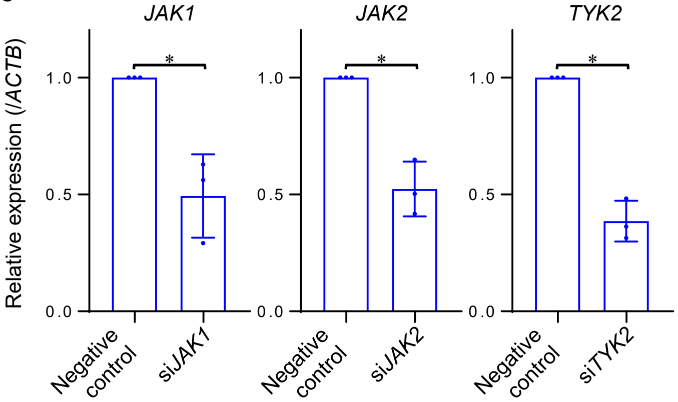

E

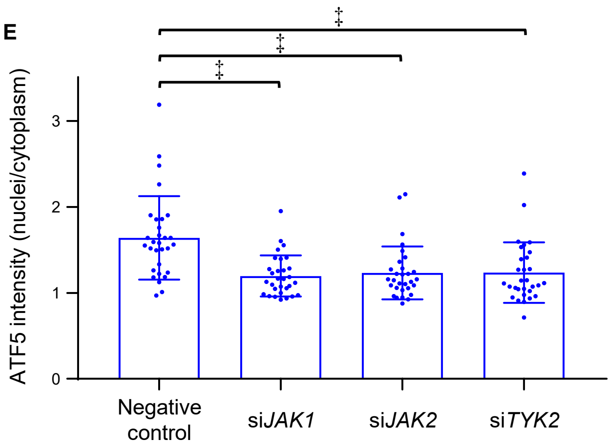

D

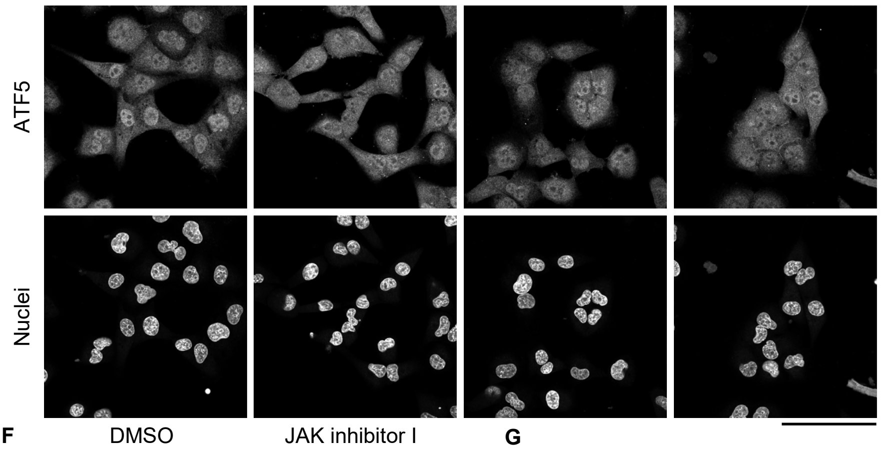

F

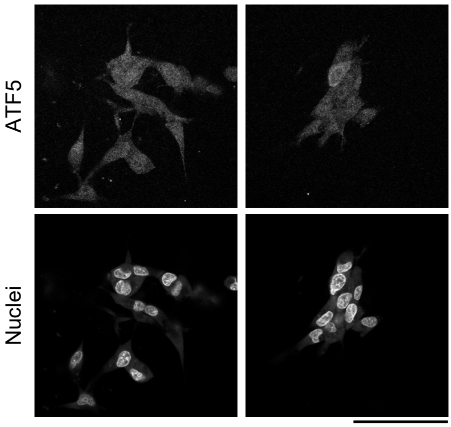

G

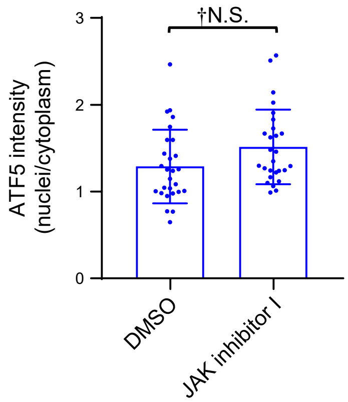

Fig. S4

Figure S4 Stiff matrix activates ATF5 via pJAK.

(A) Candidate drugs regulating ATF5 localization on collagen-coated glass plates to nuclei shown by drug screening.

(B) Western blot of pJAK and β-actin in KP4 cells treated with DMSO or JAK inhibitor I on collagen-coated plastic dishes. Relative intensity of pJAK to β-actin is shown; n = 3 experiments.

(C) qPCR of JAK family genes (*JAK1, JAK2*, or *TYK2*) in KP4 cells transfected with si*JAK1*, si*JAK2*, or si*TYK2*, respectively, on collagen-coated plastic dishes. The results of negative control RNA-transfected cells are also shown. β-actin (*ACTB*) was used as an internal control; n = 3 experiments.

(D) Immunofluorescent staining of ATF5 and nuclei in KP4 cells transfected with si*JAK1*, si*JAK2*, or si*TYK2*, respectively, on collagen-coated glass dishes.

(E) Relative intensity of ATF5 in nuclei to cytoplasm, quantified from (D); n = 27 cells in 3 experiments.

(F) Immunofluorescent staining of ATF5 and nuclei in KP4 cells treated with DMSO or JAK inhibitor I on collagen gel substrates.

(G) Relative intensity of ATF5 in the nuclei to that in the cytoplasm, quantified from (F). n = 27 cells in 2 experiments.

Scale bar = 100 μm; mean with S.D. and each data point is shown; *, statistical significance determined with 95% confidence interval; †, statistical significance ($P < 0.05$) determined via Student's t-test; ‡, statistical significance ($P < 0.05$) determined via Welch's t-test. N.S., no significance with marked test. For multiple comparisons, we analyzed significance using the Bonferroni correction.

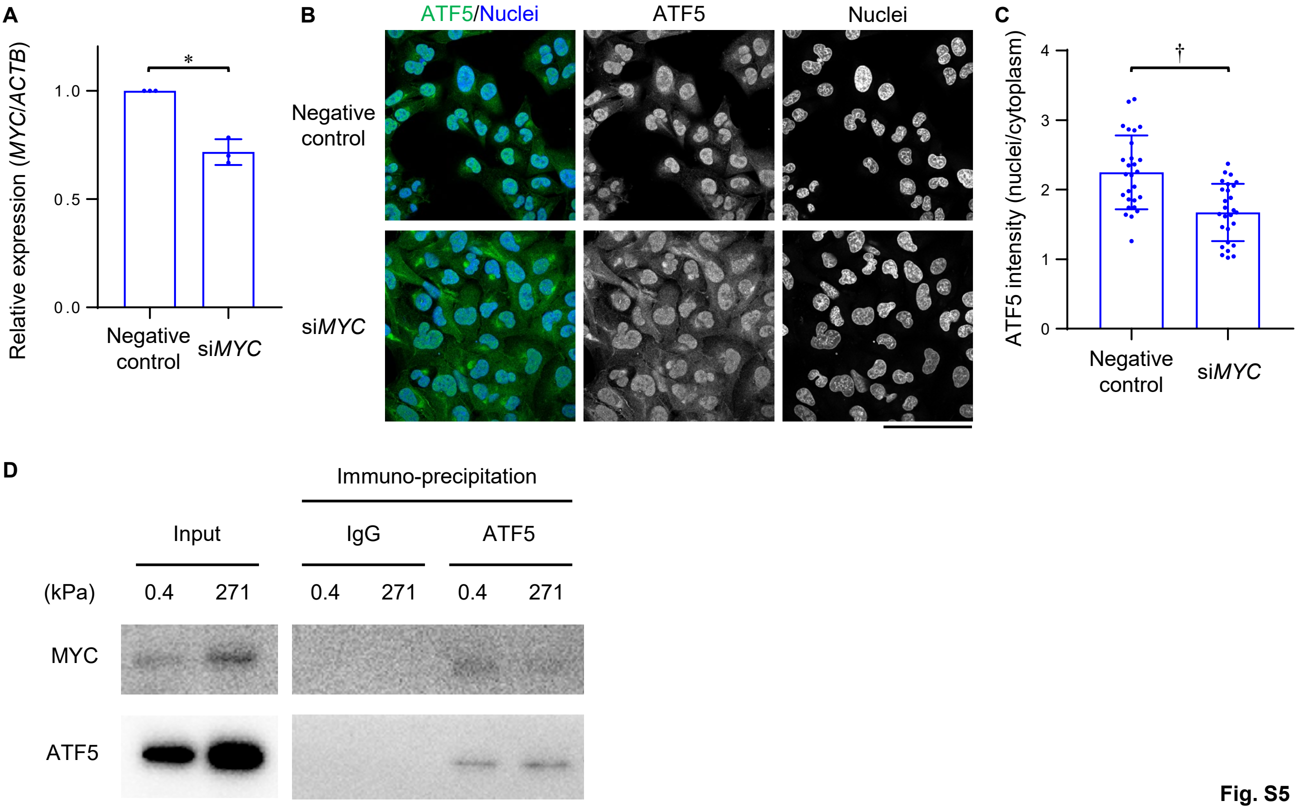

Fig. S5

Figure S5 Stiff matrix activates ATF5 via MYC.

(A) qPCR of *MYC* in KP4 cells transfected with negative control RNA or si*MYC* on collagen-coated plastic dishes. β-actin (*ACTB*) was used as an internal control; n = 3 experiments.

(B) Immunofluorescent staining of ATF5 and nuclei in KP4 cells transfected with negative control RNA or si*MYC* on collagen-coated glass dishes.

(C) Relative intensity of ATF5 in nuclei to cytoplasm quantified from (B); n = 27 cells in 3 experiments.

(D) Immunoprecipitation with control IgG or anti-ATF5 antibody followed by western blotting with anti-MYC or anti-ATF5 antibody in KP4 cells on 0.4 kPa or 271 kPa polyacrylamide gel substrates. Input sample is shown together. Representative data of 2 experiments are shown.

Scale bar = 100 μm; mean with S.D. and each data point is shown; *, statistical significance determined with 95% confidence interval; †, statistical significance ($P < 0.05$) determined via Student's t-test.

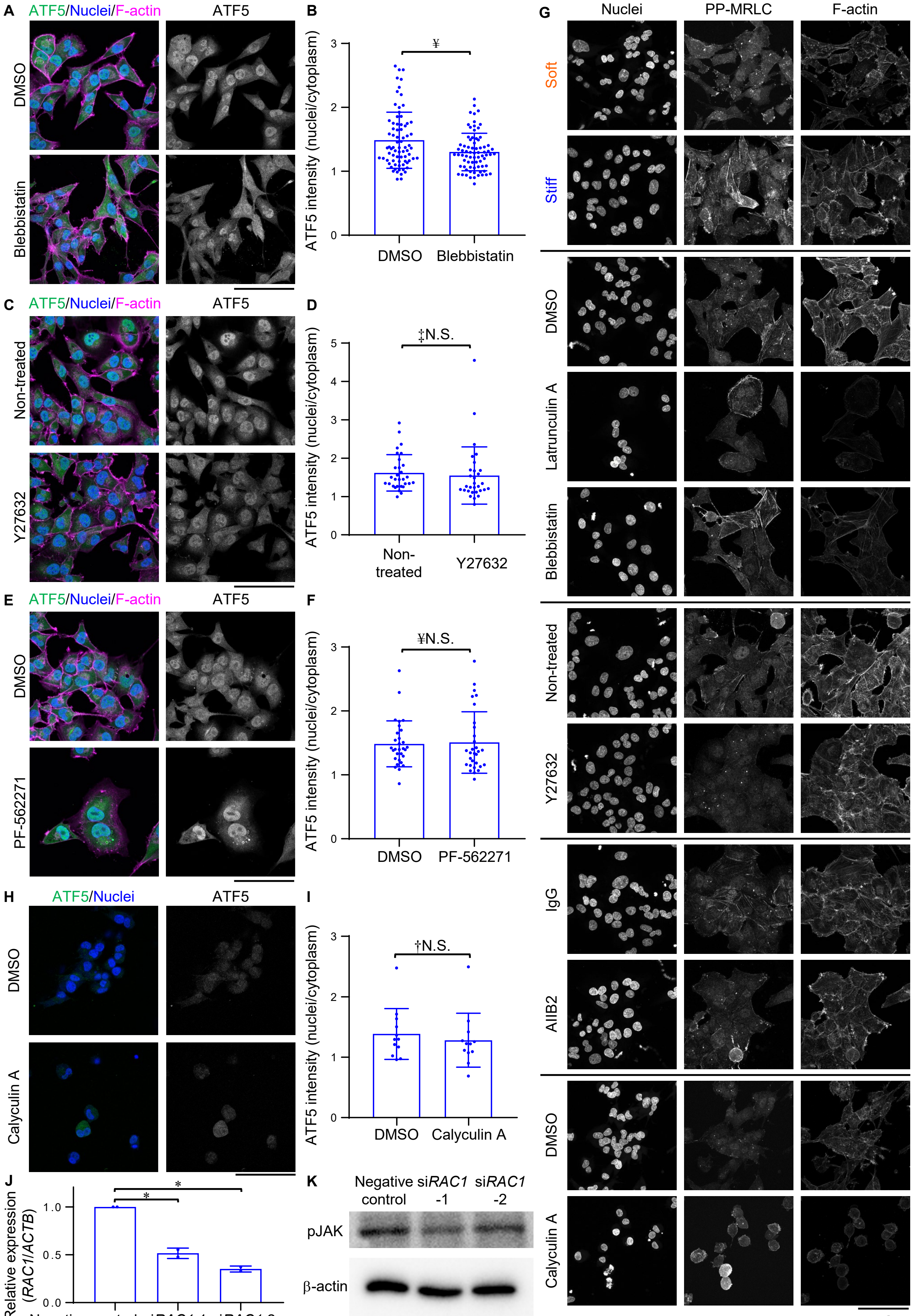

A
ATF5/Nuclei/F-actin
ATF5
DMSO
Blebbistatin
B
ATF5 intensity (nuclei/cytoplasm)
¥
DMSO
Blebbistatin
C
Non-treated
Y27632
D
‡N.S.
Non-treated
Y27632
E
DMSO
PF-562271
F
¥N.S.
DMSO
PF-562271
G
Nuclei
PP-MRLC
F-actin
Soft
Stiff
DMSO
Latrunculin A
Blebbistatin
Non-treated
Y27632
IgG
AIIB2
DMSO
Calyculin A
H
ATF5/Nuclei
DMSO
Calyculin A
I
†N.S.
DMSO
Calyculin A
J
Relative expression (RAC1/ACTB)
*
*
Negative control
siRAC1-1
siRAC1-2
K
Negative control
siRAC1 -1
siRAC1 -2
pJAK
β-actin

Fig. S6

Figure S6 ATF5 is activated in an actomyosin-dependent or independent manner.

(A) Immunofluorescent staining of ATF5, nuclei, and F-actin in KP4 cells treated with DMSO or Blebbistatin (myosin II inhibitor) on collagen-coated glass dishes.

(B) Relative intensity of ATF5 in the nuclei to that in the cytoplasm, quantified from (A); n = 75 cells in 3 experiments.

(C) Immunofluorescent staining of ATF5, nuclei, and F-actin in KP4 cells non-treated or treated with Y27632 (ROCK inhibitor) on collagen-coated glass dishes.

(D) Relative intensity of ATF5 in the nuclei to that in the cytoplasm, quantified from (C); n = 30 cells in 2 experiments.

(E) Immunofluorescent staining of ATF5, nuclei, and F-actin in KP4 cells treated with DMSO or PF-562271 (FAK inhibitor) on collagen-coated glass dishes.

(F) Relative intensity of ATF5 in the nuclei to that in the cytoplasm, quantified from (E); n = 30 cells in 2 experiments.

(G) Immunofluorescent staining of nuclei, di-phosphorylated MRLC (PP-MRLC), and F-actin in KP4 cells cultured on collagen gel (soft) or collagen-coated glass (stiff) substrates; KP4 cells treated with DMSO, Latrunculin A (actin polymerization inhibitor), or Blebbistatin on collagen-coated glass dishes; KP4 cells non-treated or treated with Y27632 (ROCK inhibitor) on collagen-coated glass dishes; KP4 cells treated with control IgG or AIIB2 (integrin β1 blocking antibody) on collagen-coated glass dishes; and KP4 cells treated with DMSO or Calyculin A (phosphatase inhibitor) on collagen gel substrates. Maximum intensity projection images from z-stack data are shown. Representative images of 2 experiments are shown.

(H) Immunofluorescent staining of ATF5 and nuclei in KP4 cells treated with DMSO or Calyculin A on collagen gel substrates.

(I) Relative intensity of ATF5 in the nuclei to that in the cytoplasm, quantified from (H); n = 12 cells in one experiment.

(J) qPCR of Rac1 (*RAC1*) in KP4 cells transfected with negative control RNA, si*RAC1*-1, or si*RAC1*-2 on collagen-coated plastic dishes. β-actin (*ACTB*) was used as an internal control; n = 2 experiments.

(K) Western blot of pJAK and β-actin in KP4 cells transfected with negative control RNA, si*RAC1*-1, or si*RAC1*-2 on collagen-coated plastic dishes. Representative blots of n = 2 experiments are shown.

Scale bar = 100 μm; mean with S.D. and each data point is shown; *, statistical significance determined with 95% confidence interval; †, statistical significance ($P < 0.05$) determined via Student's t-test; ‡, statistical significance ($P < 0.05$) determined via Welch's t-test; ¥, statistical significance ($P < 0.05$) determined via Wilcoxon rank-sum test; N.S., no significance with marked test. For multiple comparisons, we analyzed significance using the Bonferroni correction.

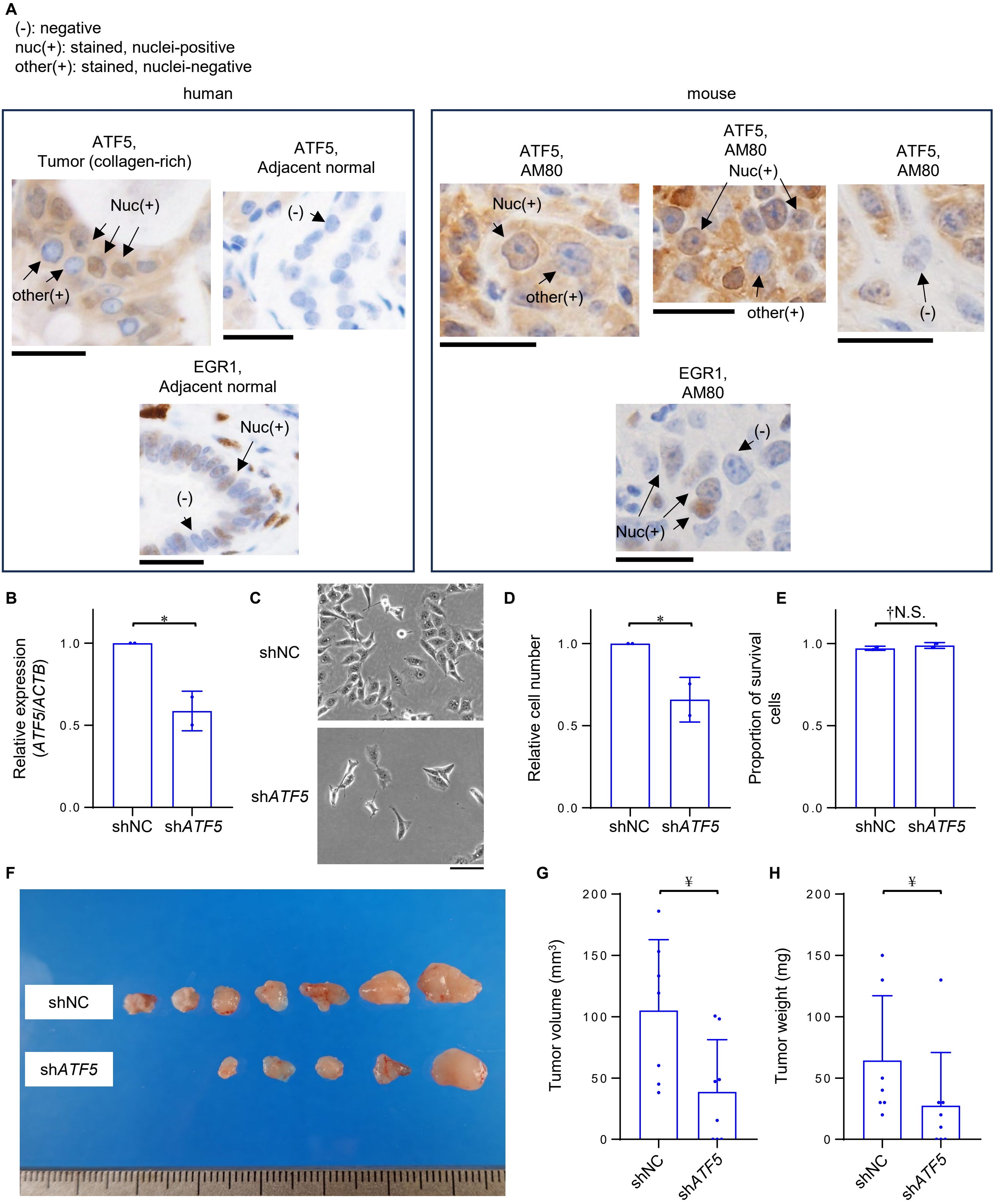
A
(-): negative
nuc(+): stained, nuclei-positive
other(+): stained, nuclei-negative
human
mouse
ATF5, Tumor (collagen-rich)
Nuc(+)
other(+)
ATF5, Adjacent normal
(-)
EGR1, Adjacent normal
Nuc(+)
(-)
ATF5, AM80
Nuc(+)
other(+)
ATF5, AM80
Nuc(+)
other(+)
ATF5, AM80
(-)
EGR1, AM80
(-)
Nuc(+)
B
Relative expression (ATF5/ACTB)
1.0
0.5
0.0
*
shNC
shATF5
C
shNC
shATF5
D
Relative cell number
1.0
0.5
0.0
*
shNC
shATF5
E
Proportion of survival cells
†N.S.
1.0
0.5
0.0
shNC
shATF5
F
shNC
shATF5
G
Tumor volume (mm³)
200
150
100
50
0
¥
shNC
shATF5
H
Tumor weight (mg)
200
150
100
50
0
¥
shNC
shATF5

Fig. S7

Figure S7 ATF5 is highly localized in the nuclei of human and mouse pancreatic cancer cells in stiff tumors.

(A) Representative images for tumor cells that are negative for ATF5 (-), nuclear positive for ATF5 (Nuc(+)), or nuclear negative and cytoplasm-positive for ATF5 (Other(+)) in tissue sections obtained from human ductal adenocarcinoma (left panels) and tumors developed in mice subcutaneously injected with a pancreatic cancer cell line mT5 and treated with the synthetic retinoid AM80 (right panels); scale bars = 25 µm.

(B) qPCR of *ATF5* in non-silencing control shRNA-transgenic (shNC) or sh*ATF5*-transgenic (sh*ATF5*) KP4 cells on collagen-coated plastic dishes. β-actin (*ACTB*) was used as an internal control; n = 2 experiments.

(C) Phase-contrast images of shNC or sh*ATF5* KP4 cells on collagen-coated plastic dishes. Scale bar = 100 µm.

(D) Relative cell number, analyzed from (C); n = 2 experiments.

(E) Proportion of surviving cells, analyzed from (C); n = 2 experiments.

(F) Tumors of shNC or sh*ATF5* KP4 cells in nude mice 30 days after injection; n = 7 (shNC) or 8 (shATF5) mice. Scale = 1 mm.

(G) Volume of tumors analyzed from (F).

(H) Weight of tumors analyzed from (F).

Mean with S.D. and each data point is shown; *, statistical significance determined with 95% confidence interval; †, statistical significance ($P < 0.05$) determined via Student's t-test; ¥, statistical significance ($P < 0.05$) determined via Wilcoxon rank-sum test; N.S., no significance with marked test.

Table S1 Clinical and demographic details of patients related to Figure 7A, B, C.

Case	Age	Sex/Gender	Race	TNM	Stage
1	54	Male	Asian (Japanese)	T3N1M0	IIB
2	73	Male	Asian (Japanese)	T3N1M0	IIB
3	77	Male	Asian (Japanese)	T3N0M0	IIA
4	77	Male	Asian (Japanese)	T3N1M0	IIB
5	68	Male	Asian (Japanese)	T3N1M0	IIB
6	80	Female	Asian (Japanese)	T3N1M0	IIB
7	62	Male	Asian (Japanese)	T3N1M0	IIB
8	75	Female	Asian (Japanese)	T3N0M0	IIA
9	63	Female	Asian (Japanese)	T3N0M1	IV
